# Liver sinusoidal endothelial cells contribute to portal hypertension through collagen type IV–driven sinusoidal remodeling

**DOI:** 10.1172/jci.insight.174775

**Published:** 2024-05-07

**Authors:** Can Gan, Usman Yaqoob, Jianwen Lu, Man Xie, Abid Anwar, Nidhi Jalan-Sakrikar, Sofia Jerez, Tejasav S. Sehrawat, Amaia Navarro-Corcuera, Enis Kostallari, Nawras W. Habash, Sheng Cao, Vijay H. Shah

**Affiliations:** 1 Department of Gastroenterology, West China Hospital, Sichuan University, Chengdu, China.; 2 Division of Gastroenterology and Hepatology, Mayo Clinic, Rochester, Minnesota, USA.; 3 Department of Hepatobiliary Surgery, the First Affiliated Hospital of Xi’an Jiaotong University, Xi’an, China.; 4 Affiliated Hospital of Qingdao University, Qingdao, China.

**Keywords:** Cell biology, Hepatology, Collagens, Endothelial cells

## Abstract

Portal hypertension (PHTN) is a severe complication of liver cirrhosis and is associated with intrahepatic sinusoidal remodeling induced by sinusoidal resistance and angiogenesis. Collagen type IV (COL4), a major component of basement membrane, forms in liver sinusoids upon chronic liver injury. However, the role, cellular source, and expression regulation of COL4 in liver diseases are unknown. Here, we examined how COL4 is produced and how it regulates sinusoidal remodeling in fibrosis and PHTN. Human cirrhotic liver sample RNA sequencing showed increased COL4 expression, which was further verified via immunofluorescence staining. Single-cell RNA sequencing identified liver sinusoidal endothelial cells (LSECs) as the predominant source of COL4 upregulation in mouse fibrotic liver. In addition, COL4 was upregulated in a TNF-α/NF-κB–dependent manner through an epigenetic mechanism in LSECs in vitro. Indeed, by utilizing a CRISPRi-dCas9-KRAB epigenome-editing approach, epigenetic repression of the enhancer-promoter interaction showed silencing of COL4 gene expression. LSEC-specific COL4 gene mutation or repression in vivo abrogated sinusoidal resistance and angiogenesis, which thereby alleviated sinusoidal remodeling and PHTN. Our findings reveal that LSECs promote sinusoidal remodeling and PHTN during liver fibrosis through COL4 deposition.

## Abstract 2

Liver sinusoidal endothelial cells promote sinusoidal remodeling during fibrosis via generation of collagen type IV, subsequent basement membrane formation, and angiogenic activation.

## Graphical abstract

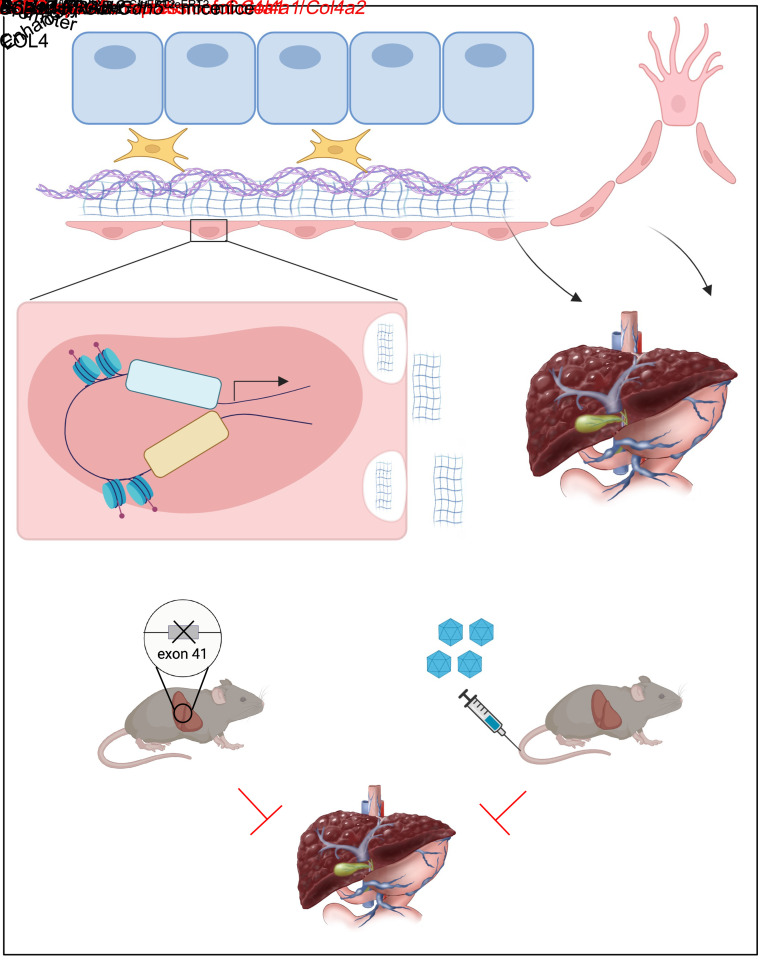

## Introduction

Portal hypertension (PHTN) is a consequence of liver cirrhosis and a leading cause of liver transplant and death in patients with cirrhosis (1). According to the hydraulic equivalent of Ohm’s law, portal pressure is determined by blood flow and resistance. Therefore, the pathophysiology of PHTN can be attributed to increased blood flow, increased vascular resistance, or a combination of both (2). Liver sinusoidal endothelial cells (LSECs), which form the permeabilized barrier of liver sinusoids, are critical regulators of hepatic microcirculation and portal pressure (3). LSECs have been shown to initiate sinusoidal remodeling during the progression of PHTN (1). When exposed to liver injury, liver sinusoids are remodeled, with the loss of LSEC fenestrae and formation of an organized basement membrane (a process termed capillarization) (4), as well as sinusoidal angiogenesis (5). Capillarized sinusoids with basement membrane formation contribute to sinusoidal stiffness, which leads to increased hepatic vascular resistance and the development of PHTN (1). Meanwhile, the capillarized LSECs have the phenotype of common endothelial cells, which can form new blood vessels from preexisting vascular beds, a process termed angiogenesis (6, 7). Increased blood flow caused by angiogenesis in the intrahepatic circulation contributes to PHTN. However, the mechanisms underlying sinusoidal remodeling are not well understood.

Inflammatory signaling also contributes to PHTN through effects on sinusoidal remodeling (5). Prior publications from our group and others showed that inflammatory stimulation (8, 9), including that by TNF-α, leads to the loss of the LSEC phenotype (9) and to subsequent aberrant angiocrine signaling, which recruits immune cells to liver sinusoids (10–14). Inflammatory stimulation by lipopolysaccharides promotes the activation and invasion of LSECs, which contributes to angiogenesis and PHTN (15). Dysfunctional LSECs after inflammatory insult remodel liver sinusoids by promoting hepatic vascular resistance and angiogenesis during the development of PHTN (8, 16). However, the mechanisms by which inflammatory signaling leads to downstream transcriptional regulation in sinusoidal remodeling remain unknown.

Epigenetic mechanisms, such as posttranslational modifications of histone, are involved in transcriptional regulation in human health and disease. Histone marks indicate the activity of the target gene or regulatory elements (17). For example, the research on liver fibrosis shows that trimethylation of histone H3 at lysine 4 (H3K4me3) is common at the promoter region of an active gene, and single methylation of histone H3 at lysine 4 (H3K4me1) is seen at enhancer regions, whereas histone H3 acetylation at lysine 27 (H3K27ac) is noted at both enhancer and promoter regions of active genes (18). Epigenome editing is a way to manipulate gene expression without editing the DNA sequences. The epigenome-editing approach CRISPRi-dCas9-KRAB (clustered regularly interspaced short palindromic repeat interference [CRISPRi], based on the fusion of nuclease-deactivated Cas9 [dCas9] to the Krüppel-associated box [KRAB]) has become a widespread method to manipulate targeted gene expression because of its high efficiency, specificity, and ease of use (19–21). dCas9 fusion with the epigenetic repressor domain KRAB (dCas9-KRAB) recruits histone methyltransferases (e.g., for histone 3 lysine 9 trimethylation [H3K9me3]) to the enhancer or promoter region of the targeted gene under the guidance of single-guide RNA (sgRNA) to promote a regressive effect (22). Prior work from our group has shown the efficiency and mechanism of dCas9-KRAB on repressing chemokine expression via recruiting histone marks to the super enhancer and promoter regions of genes in vitro (12, 14). Given that dCas9-KRAB manipulates gene expression without editing DNA sequences, recent studies focus on this epigenome-editing technology and its potential clinical application. However, the efficiency of the dCas9-KRAB system in vivo, as well as in determining targeted gene function in vivo, remain unclear.

Basement membrane — network-forming extracellular matrix (ECM) between endothelial cells and the underlying interstitial tissue — provides a scaffold supporting the surrounding cells and tissues (23). Interestingly, healthy liver sinusoids lack basement membrane, which permits sinusoidal crosstalk. However, sinusoidal basement membrane becomes noticeable upon chronic liver injury. The formation of basement membrane underneath LSECs impedes cellular communication and aggravates liver dysfunction. Collagen type IV (COL4), the major component of basement membrane, has an integral role in its formation and vascular homeostasis (24). The cell type responsible for COL4 production in the liver is unclear, as are the regulation and function of COL4 in the context of liver injury.

In this study, we identified a significant relationship between COL4 deposition and the severity of PHTN. Through single-cell RNA-sequencing (scRNA-Seq) studies, we show that LSECs produce COL4, which is under the regulation of the TNF-α/NF-κB signaling axis. Moreover, epigenetic repression of enhancer-promoter interactions silences COL4 gene expression via a CRISPRi-dCas9-KRAB approach. Furthermore, in mice with COL4 gene mutation or CRISPRi-dCas9-KRAB–mediated COL4 gene repression, we show that LSEC-derived COL4 contributes to the development of PHTN. Mechanistic studies indicate that COL4 promotes sinusoidal resistance through LSEC capillarization and basement membrane formation, as well as by supplying a platform for collagen type I (COL1) binding and assembly. Moreover, COL4 contributes to sinusoidal angiogenesis by activating angiogenic sprouting of LSECs. Taken together, our results indicate a critical role for LSEC-derived COL4 in sinusoidal remodeling and PHTN.

## Results

### COL4 is upregulated in human and mouse fibrotic livers.

Liver cirrhosis and its complication PHTN are associated with the formation of ECM, for which collagens are the core components that provide structural support. Several types of collagens are involved in liver fibrosis and PHTN (25). Of these, fibril-forming COL1 is the most abundant during liver fibrosis (26, 27). Network-forming COL4 — a heterotrimer containing 2 α1 chains and 1 α2 chain — is the main constituent of basement membrane of the vessels and is noted in fibrotic livers (24).

**Figure 1:**
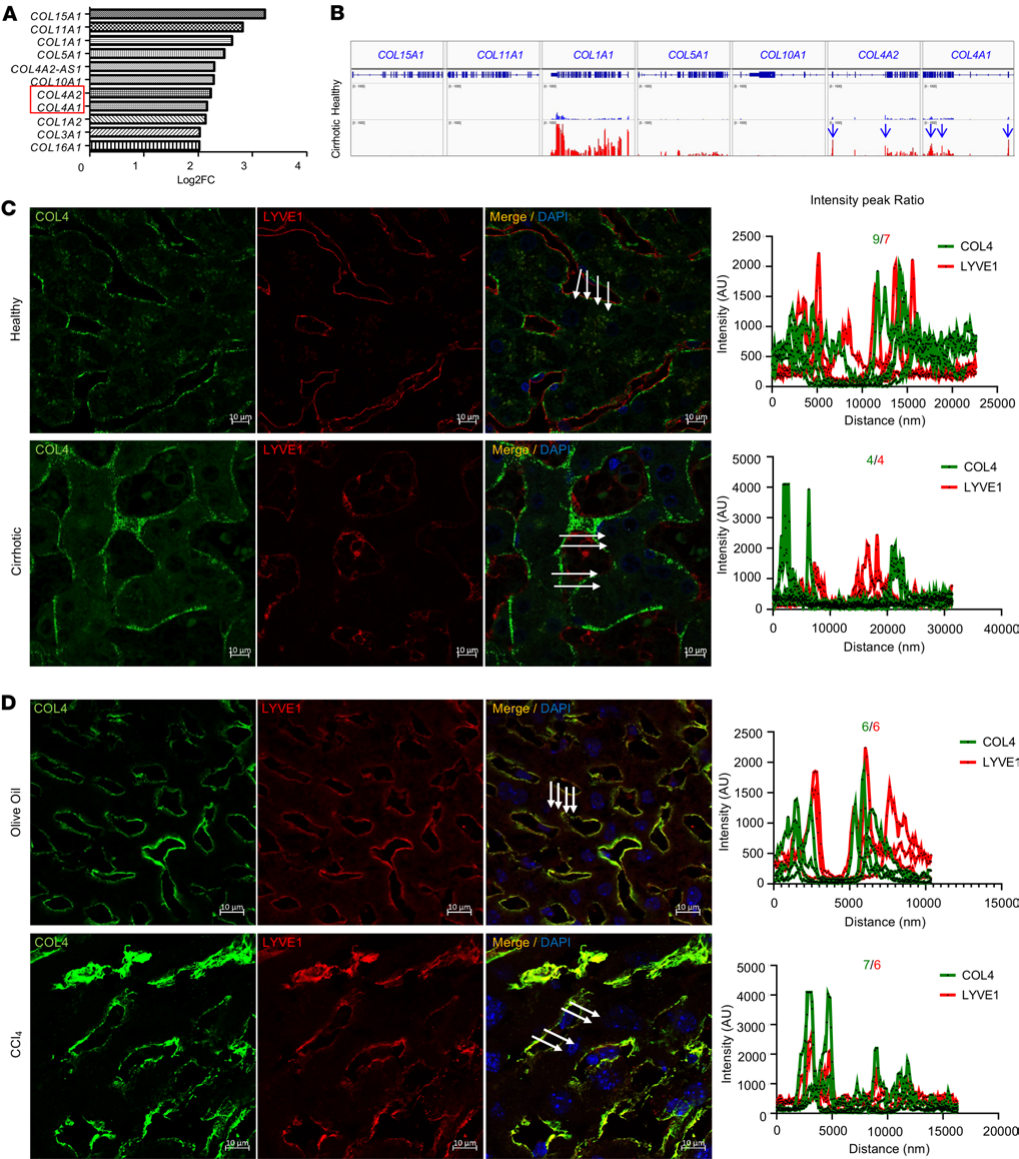
COL4 is increased in human and mouse fibrotic livers. (**A**) Differential gene expression analysis showed a wide array of collagen genes with increased expression (log_2_ fold-change [Log_2_FC] > 2) in alcohol-induced human cirrhotic livers. Red box highlights COL4 genes. (**B**) Expression level of upregulated collagen genes in healthy and cirrhotic human livers was quantified via Integrative Genomics Viewer. Other than *COL1A1*, both *COL4A1* and *COL4A2* (white arrows) showed the highest reads per kilobase per million mapped reads (RPKM) in cirrhotic livers. (**C** and **D**) Representative immunofluorescence (IF) images showed COL4 staining (green) and LYVE1 staining (red) in human (**C**) and mouse (**D**) livers. Beside each image is the colocalization analysis for each of the merged images. The graph peaks represent the average fluorescence intensities of the staining at the given distances (in nanometers) along the 4 straight white arrows in each sinusoid. The white arrows in the merged images represent the areas selected for colocalization. Each graph has number of intensity peaks for each fluorophore (green and red). The ratio of intensity peaks of COL4 versus LYVE1 is shown on the top of each graph. A total of 3 complete sinusoids have been quantified for colocalization analysis (*n* = 3/group). IF images showed close approximation of COL4 with Lyve1 in both human and mouse normal and cirrhotic liver. DAPI (blue) was used to stain nuclei. COL4 expression was upregulated in liver sinusoids from alcohol-induced human cirrhotic livers (**C**) and from CCl_4_-induced mouse fibrotic livers (**D**). Scale bars: 10 μm. Graphs represent mean ± SEM. P < 0.05 is for the comparison of panel **C** IF intensity from healthy to cirrhotic. *P* < 0.01 is for the comparison of panel **D** IF intensity from olive oil to CCL_4_.

To examine changes in collagen expression in liver samples of patients with PHTN, we performed RNA-Seq analysis of human alcohol-induced cirrhotic and normal livers. We identified 950 upregulated genes and 761 downregulated genes in human cirrhotic livers compared with normal livers (Supplemental Figure 1; supplemental material available online with this article; https://doi.org/10.1172/jci.insight.174775DS1). Among the differentially expressed genes, a wide array of collagen genes showed increased expression, of which *COL1A1*, *COL4A1*, and *COL4A2* (the genes for the COL1 α1 and COL4 α1 and α2 chains, respectively) were most notably overexpressed in cirrhotic livers (Figure 1A). To better quantify the amounts of these collagens, we then used Integrative Genomics Viewer software (Broad Institute) to visualize the normalized expression level of these collagens in healthy and cirrhotic livers (Figure 1B). Other than *COL1A1*, *COL4A1* and *COL4A2* had some of the highest RPKM in cirrhotic livers. These data reveal that *COL4A1* and *COL4A2* are not only the most upregulated collagen genes but also 2 of the top expressed collagens in cirrhotic liver. To validate our observation, we performed immunofluorescence (IF) costaining with COL4 and LSEC marker LYVE1. This showed increased COL4 expression in human cirrhotic livers compared with healthy livers (Figure 1C). COL4-positive areas in liver sinusoids were near LYVE1-positive areas in both human healthy and cirrhotic livers. The peak intensities and number of peaks and ratio for COL4 and LYVE1 were approximately equal. Consistent with the results in human livers, we noted a similar increase in COL4 expression in a carbon tetrachloride–induced (CCl_4_-induced) mouse model of liver fibrosis (Figure 1D). COL4 was highly overlapped with LYVE1-positive areas in both healthy and fibrotic livers. Taken together, these data indicate that LSEC-derived COL4 is upregulated with the development of liver fibrosis in human and mouse livers.

### LSECs are the primary source of COL4 in healthy and fibrotic livers.

**Figure 2:**
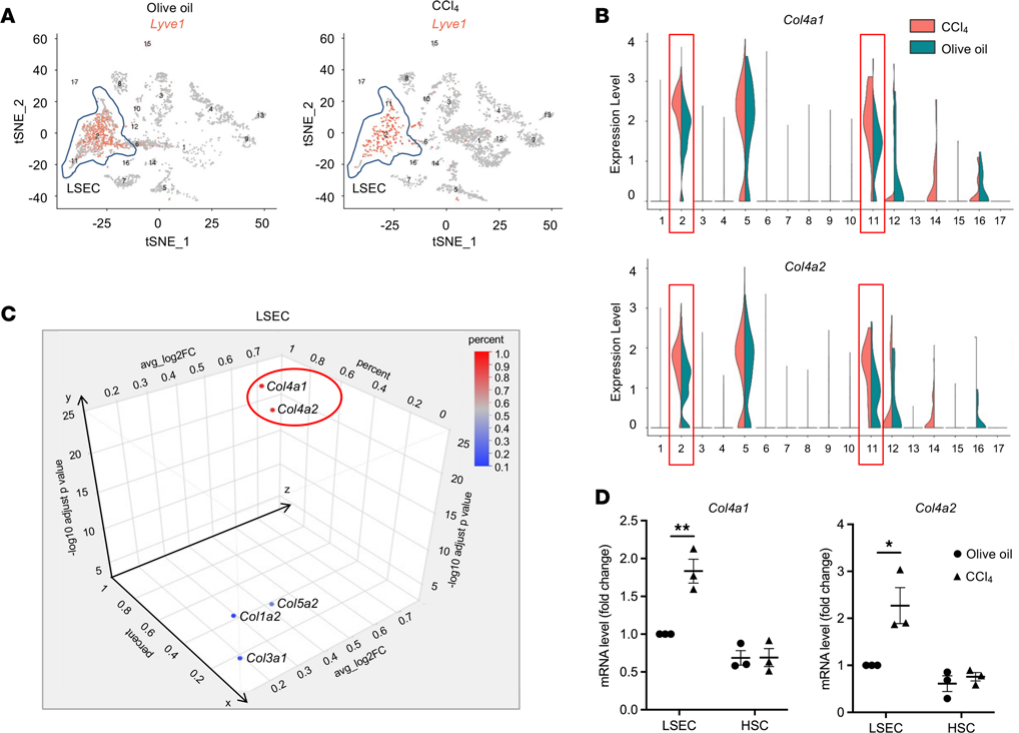
LSECs are the main source of *Col4a1* and *Col4a2* in healthy and pathologic conditions. Single-cell RNA-sequencing (scRNA-Seq) analysis was performed on normal (olive oil) and fibrotic (CCl_4_) mouse livers. (**A**) t-Distributed stochastic neighbor embedding (t-SNE) analysis identified 17 main cell clusters (numbered) in the liver using distinct gene markers. Among them, *Lyve1* was a marker for LSECs, which represent clusters 2 and 11. Each orange dot represents an individual LSEC. Other cell clusters were also identified based on the conserved genes of each cell type. Clusters 1, 4, 9, and 13 were identified as hepatocytes; cluster 5 as HSCs; cluster 3 as Kupffer cells; clusters 10 and 12 as macrophages; and cluster 16 as cholangiocytes. The rest of clusters represent other immune cells, including T cells, B cells, and neutrophils. (**B**) Violin plots for scRNA-Seq data from the 17 cell clusters show that *Col4a1* and *Col4a2* were mainly expressed by LSECs, and the expressions were higher in fibrotic livers. (**C**) By analyzing percentage of cells expressing the gene, log_2_FC of gene in fibrotic versus healthy livers, and adjusted *P* < 0.05 (adjusted *P* value from DESeq2), the 3D plot showed increased *Col4a1* and *Col4a2* in LSEC clusters in mouse fibrotic livers. (**D**) By quantitative real-time polymerase chain reaction (qPCR) assays of isolated LSECs and HSCs from mouse normal and fibrotic livers, LSECs, but not HSCs, from fibrotic livers showed markedly increased *Col4a1* and *Col4a2* gene expression (*n* = 3/group). Graphs represent mean ± SEM. *P < 0.05, **P < 0.01, 2-tailed unpaired *t* test.

Given the increased expression of COL4 in fibrotic livers, we sought to determine the predominant cell type producing COL4 in the liver and assess changes in its expression during the development of liver fibrosis. We performed scRNA-Seq analysis using samples from normal and CCl_4_-induced fibrotic mouse livers. All cell types were identified based on previously published conserved genes (28). Analysis using t-distributed stochastic neighbor embedding (t-SNE) showed 17 clusters, which corresponded to hepatocytes, endothelial cells, hepatic stellate cells (HSCs), cholangiocytes, Kupffer cells, and other immune cells. Here, *Lyve1* (Figure 2A) was a marker for LSECs, and *Pdgfra* and *Pdgfrb* were markers for HSCs (Supplemental Figure 2A). Therefore, clusters 2 and 11 were identified as LSECs, and cluster 5 was identified as HSCs. We then verified that *Col4a1* and *Col4a2* were mainly expressed by LSECs and HSCs. Furthermore, LSEC-derived *Col4a1* and *Col4a2* expression levels were higher after CCl_4_ administration per violin plots, which was not the case for HSCs (Figure 2B).

LSECs account for 20% of liver-resident cells (29), which is more plentiful than the 5% of HSCs among liver-resident cells (30). Therefore, we analyzed the percentage of cells expressing COL4 gene in fibrotic mouse livers, log_2_ fold-change of gene expression in fibrotic versus healthy mouse livers, and adjusted *P* < 0.05. This method identified *Col1a1* and *Col3a1* as the most increased in HSCs (Supplemental Figure 2, B and C) and *Col4a1* and *Col4a2* as the most upregulated in LSECs (Figure 2C). In contrast, we observed that sinusoidal COL4, which displayed a continuous staining pattern within the sinusoids, was near α–smooth muscle actin (α-SMA), which exhibited a scattered staining pattern around the sinusoids. However, there was no significant colocalization between the two in normal human or fibrotic liver tissues (Supplemental Figure 2, E and F). Similarly, PDGFRβ staining exhibited a scattered pattern around the sinusoidal COL4. The peak patterns and numbers for α-SMA and PDGFRβ were different compared with sinusoidal COL4. This observation suggests that COL4 production is primarily attributed to LSECs rather than HSCs. In addition, analysis of public data sets revealed that LSECs were the main cellular source of COL4 in the human liver (Supplemental Figure 3A, FANTOM5 database; https://fantom.gsc.riken.jp/zenbu/gLyphs/#config=FANTOM5_promoterome_hg19;loc=hg19:chr10:104151762.104164391+), as well as in the mouse liver (Supplemental Figure 3, B and C) (31), and accounted for COL4 upregulation in a murine nonalcoholic steatohepatitis model (Supplemental Figure 3D) (32). These public data are consistent with our scRNA-Seq findings.

We then further verified the contribution of LSECs and HSCs to COL4 expression during liver fibrosis and PHTN progression using primary LSECs and HSCs isolated from mice injected with olive oil or CCl_4_. As compared with LSECs from the olive oil–injected control, the expression of *Col4a1* and *Col4a2* was increased in LSECs from CCl_4_-injected fibrotic livers (Figure 2D). However, the expression of *Col4a1* and *Col4a2* in HSCs did not change, which is consistent with our scRNA-Seq data (Figure 2B). In summary, LSECs are the predominant cell type producing COL4 in healthy and fibrotic livers.

### TNF-α induces COL4 expression in an NF-κB–dependent manner.

**Figure 3:**
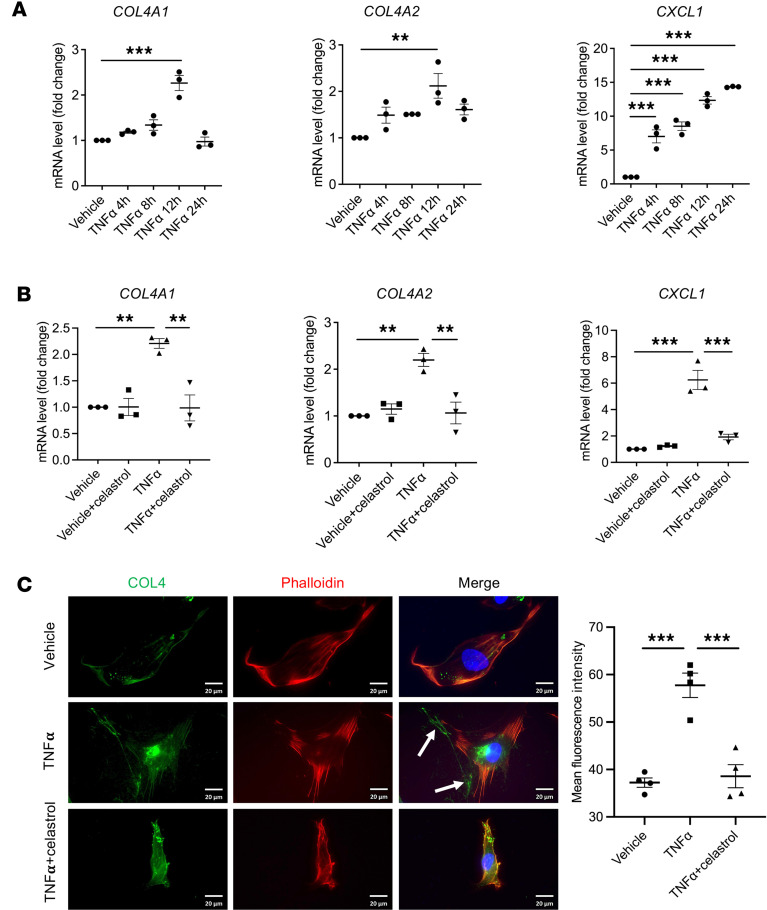
TNF-α induces expression of *COL4A1* and *COL4A2* in an NF-κB–dependent manner. (**A**) qPCR assays were performed on human LSECs treated with vehicle or TNF-α from 4 to 24 hours. mRNA levels of *COL4A1* and *COL4A2* were highest after 12 hours of TNF-α treatment. *CXCL1* was a positive control (*n* = 3, biologically independent samples). (**B** and **C**) Human LSECs were pretreated with the NF-κB inhibitor celastrol before 12 hours of TNF-α stimulation. Celastrol strikingly blocked the increase in *COL4A1*, *COL4A2*, and *CXCL1* mRNA levels (**B**) and COL4 protein (**C**) induced by TNF-α stimulation (*n* = 3–4, biologically independent samples). Green indicates COL4 stained with anti-COL4 antibody; red, phalloidin staining of actin filaments; blue, DAPI staining of DNA; white arrows, secreted COL4 protein. Scale bars: 20 μm. Graphs represent mean ± SEM. **P < 0.01, ***P < 0.001, 1-way ANOVA followed by Bonferroni’s posttest.

Based on the transcriptional differences from scRNA-Seq, we next investigated the transcriptional regulation of *COL4A1* and *COL4A2* in LSECs. RNA-Seq data of human cirrhotic liver from our group identified TNF-α as a top upstream regulator by Ingenuity Pathway Analysis (QIAGEN) (Supplemental Figure 4). Given that *COL4A1* and *COL4A2* were among the most upregulated collagens in human cirrhotic livers (Figure 1A), we hypothesized that COL4 is regulated by TNF-α and has a role in liver fibrosis and PHTN. To test this hypothesis, we treated human LSECs with TNF-α and verified a substantial increase in mRNA expression of *COL4A1* and *COL4A2* (Figure 3A) in response to TNF-α stimulation. NF-κB is an important transcription factor downstream of inflammatory stimuli, including TNF-α (33). Analysis of public chromatin immunoprecipitation-sequencing (ChIP-Seq) data sets showed that NF-κB occupied the bidirectional promoter region and putative enhancer regions of *COL4A1/COL4A2* in human umbilical vein endothelial cells after TNF-α treatment (Supplemental Figure 5). To assess the potential role of TNF-α/NF-κB signaling in COL4 expression, we pretreated human LSECs with celastrol, an NF-κB inhibitor. Celastrol strikingly blocked the effect of TNF-α and abrogated the expression of *COL4A1* and *COL4A2* at both the mRNA and protein level (Figure 3, B and C). These findings indicate that TNF-α upregulates production of COL4 in LSECs in an NF-κB–dependent manner.

### Epigenetic repression of the enhancer-promoter interaction silences Col4a1 and Col4a2 gene expression.

The enhancer-promoter interaction is the linking of distal enhancers with proximal promoters by chromatin looping; these regions harbor transcription factors, coactivators, RNA polymerase II, and the Mediator complex, to orchestrate gene expression (18, 34). Histone mark signals are reliable indicators of active enhancers and promoters (18, 35). Enhancer activity is critical for cell type–specific and spatiotemporal expression of genes (36). To identify the putative enhancer region of LSEC *Col4a1/Col4a2*, we performed tagmentation-assisted fragmentation of ChIP-Seq (TAF–ChIP-Seq) of transcription activation mark H3K27ac on isolated mouse LSECs and then speculated 5 putative enhancer regions in H3K27ac-occupied loci (Supplemental Figure 6A, red rectangles). Interestingly, *Col4a1/Col4a2* gene loci that were enriched with H3K27ac and H3K4me1 in mouse livers from public ChIP-Seq data sets, both of which were marks of enhancer regions, highly overlapped with the 5 speculated enhancer regions in our LSEC TAF–ChIP-Seq data (Supplemental Figure 6B). Moreover, these gene loci showed increased H3K27ac occupancy at both the putative enhancer and promoter regions in human cirrhotic liver (Supplemental Figure 7), indicating that transcription of *Col4a1/Col4a2* is activated in cirrhotic liver. Based on the above histone modification marks, these findings identify potentially novel enhancers and a promoter of COL4 genes, which might be targets to regulate COL4 gene expression and to investigate the role of COL4 in liver fibrosis and PHTN without editing DNA sequences.

**Figure 4:**
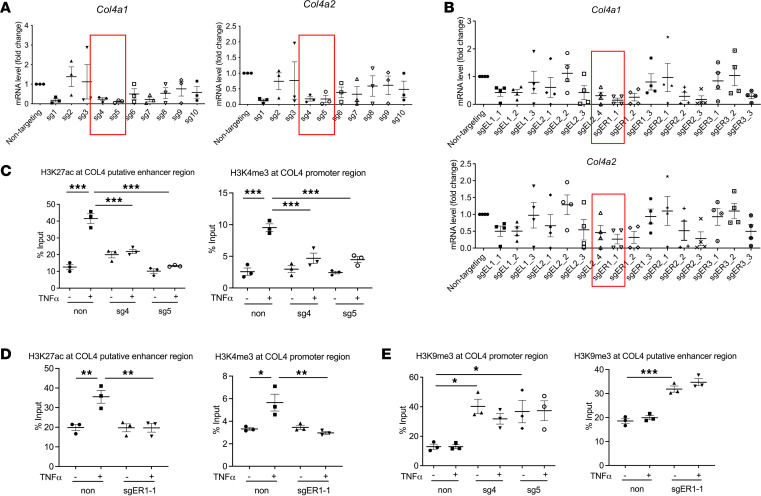
Epigenetic repression of the enhancer-promoter interaction silences *Col4a1* and *Col4a2* gene expression in response to TNF-α stimulation. Isolated LSECs from *dCas9-KRAB Cdh5*^CreERT2^ mice after in vivo tamoxifen injection were transfected with single-guide RNAs (sgRNAs), which target the bidirectional promoter region or putative enhancer regions of the COL4 genes. Nontargeting sgRNA was used as a negative control. (**A**) Among sgRNAs targeting the bidirectional promoter region, sgRNAs 4 (sg4) and 5 (sg5) most strongly repressed the expression of *Col4a1* and *Col4a2* (red boxes) compared with nontargeting sgRNA via qPCR assay (*n* = 3, biologically independent samples). (**B**) Among sgRNAs targeting the putative enhancer regions, sgER1-1 (red boxes) most strongly repressed the expression of *Col4a1* and *Col4a2* compared with nontargeting sgRNA via qPCR (*n* = 4, biologically independent samples). (**C**) ChIP-qPCR assay revealed decreased H3K4me3 enrichment at the promoter region, as well as decreased H3K27ac enrichment at the putative enhancer region, after sg4 and sg5 transfection into isolated mouse LSECs expressing dCas9-KRAB (*n* = 3, biologically independent samples). (**D**) ChIP-qPCR showed decreased H3K27ac enrichment at the putative enhancer region, as well as decreased H3K4me3 enrichment at the promoter region, after sgER1-1 was transfected into isolated mouse LSECs expressing dCas9-KRAB (*n* = 3, biologically independent samples). (**E**) ChIP-qPCR showed increased H3K9me3 enrichment at the corresponding region when sgRNAs targeted the promoter or putative enhancer regions (*n* = 3, biologically independent samples). Graphs represent mean ± SEM. *P < 0.05, **P < 0.01, ***P < 0.001, 1-way ANOVA followed by Bonferroni’s posttest.

Next, we aimed to explore if disturbing enhancer-promoter interaction could regulate COL4 gene expression and the potential mechanisms. For this, we utilized the CRISPRi-dCas9-KRAB epigenome editing approach. dCas9-KRAB recruits H3K9me3 together with heterochromatin protein 1 to induce heterochromatin, chromatin that is inaccessible to the binding of transcriptional regulators. To find the exact loci in the enhancer and the promoter regions that affect COL4 expression, we used Benchling software (14) to design sgRNAs targeting different loci of *Col4a1/Col4a2*: 10 sgRNAs targeting the bidirectional promoter region and 16 sgRNAs targeting the putative enhancer regions (Supplemental Figure 8A). LSECs were isolated from *dCas9-KRAB Cdh5*^CreERT2^ (vascular endothelial cadherin promoter with a CreERT2 coding sequence) mice after in vivo tamoxifen administration to induce dCas9-KRAB expression specifically in LSECs (Supplemental Figure 8B). The isolated LSECs were transfected with individual synthetic sgRNAs targeting COL4 promoter. *Col4a1* and *Col4a2* expression was significantly decreased with sg4 and sg5 compared with nontargeting sgRNA (Figure 4A). The expression of the endothelial cell marker *Pecam1*, as a control, was unchanged (Supplemental Figure 8C). In addition, sgRNA ER1_1 targeting the putative enhancer region showed the greatest repression of *Col4a1/Col4a2*, whereas the expression of *Pecam1* was unchanged (Figure 4B and Supplemental Figure 8D).

To verify the enrichment of histone marks at the enhancer and promoter regions induced by dCas9-KRAB, we performed ChIP-qPCR. The targeting of the promoter region by sgRNAs decreased H3K4me3 enrichment at the promoter region and decreased H3K27ac enrichment at the enhancer region, which suggests that disturbing the promoter interferes with enhancer activity (Figure 4C). Likewise, the targeting of the putative enhancer region by sgRNAs decreased H3K27ac enrichment at the enhancer region and H3K4me3 enrichment at the promoter region (Figure 4D). This indicates that these regions are likely the enhancers for *Col4a1/Col4a2* transcription and that the enhancer interacts with the promoter for gene transcription (Figure 4D). To verify that this effect of dCas9-KRAB is site specific, we performed ChIP-qPCR and found that H3K9me3 occupied corresponding sites when sgRNA targeted the promoter or enhancer region (Figure 4E). These results demonstrate that epigenetic repression of the enhancer-promoter interaction by the epigenome-editing approach silences COL4 gene expression with high efficacy and specificity in vitro, which paves the road for epigenome editing of transcriptional regulatory elements of COL4 genes in vivo and shows the therapeutic potential of COL4 repression in liver fibrosis and PHTN.

### LSEC-specific COL4 contributes to PHTN.

**Figure 5:**
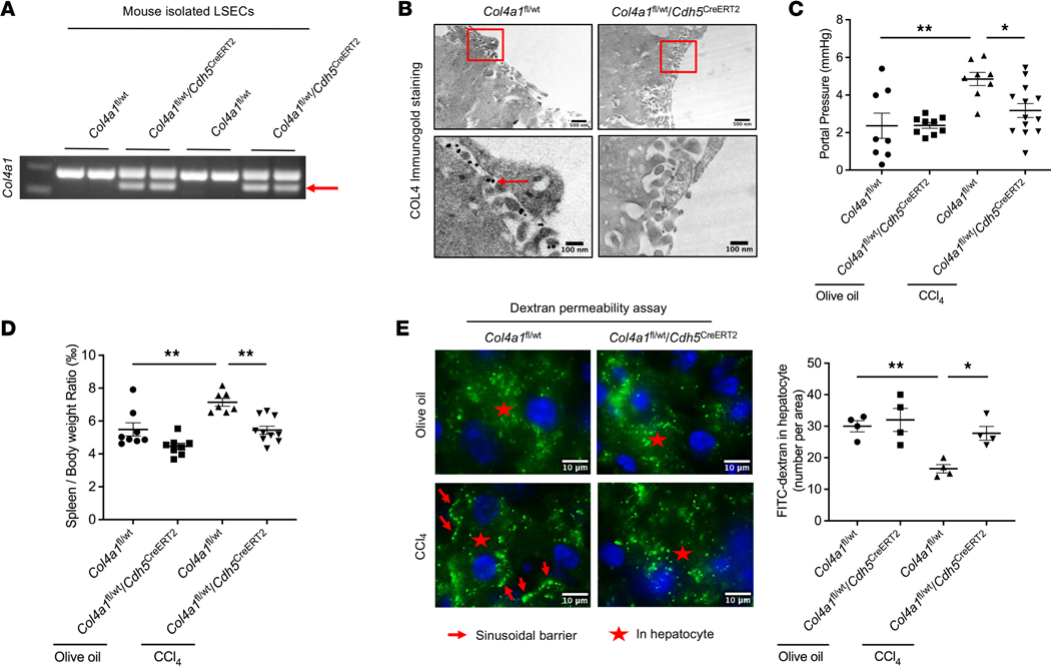
LSEC-specific COL4 contributes to PHTN. (**A**) LSEC-specific *Col4a1*-mutant mice were generated by crossing *Col4a1*^fl/wt^ mice with *Cdh5*^CreERT2^ mice. *Col4a1* mutation was induced by deleting exon 41 to produce a truncated dysfunctional COL4 protein. LSECs were isolated from *Col4a1*^fl/wt^ and *Col4a1*^fl/wt^ *Cdh5*^CreERT2^ mice after in vivo tamoxifen administration to extract DNA for PCR. Post-PCR gel showed a lower–molecular weight band after Cre recombinase treatment to excise the floxed exon 41 sequence in *Col4a1*^fl/wt^ *Cdh5*^CreERT2^ mouse LSECs (red arrow). (**B**) COL4 immunogold staining of mouse liver tissues showed that COL4 protein was secreted and deposited in liver sinusoids from *Col4a1*^fl/wt^ mice (red arrow), whereas LSEC-specific *Col4a1* mutation inhibited COL4 deposition. Bottom images are 100 nm magnifications of red boxes on top 500 nm images. (**C**) To induce PHTN, *Col4a1*^fl/wt^ and *Col4a1*^fl/wt^ *Cdh5*^CreERT2^ mice were subjected to 6 weeks of CCl_4_ administration (or olive oil for control). CCl_4_ injection increased portal pressure by 2-fold in *Col4a1*^fl/wt^ mice, whereas this increase was abrogated by *Col4a1* mutation in LSECs (*n* = 8–13/group). (**D**) The ratio of spleen weight to body weight was increased after CCl_4_ administration, which was blocked by LSEC *Col4a1* mutation (*n* = 7–10/group). (**E**) To explore liver sinusoidal permeability, 4 kDa FITC-dextran was administered via the tail vein. It passed through LSECs and was endocytosed by hepatocytes (red stars) in healthy livers. In contrast, FITC-dextran failed to cross the membrane of CCl_4_-induced LSECs, gathering at the sinusoidal line (red arrows). FITC-dextran was noticeable in hepatocytes from *Col4a1*-mutated livers even with CCl_4_ treatment, which indicates that COL4 blocks sinusoidal permeability (*n* = 4/group). Scale bars: 10 μm. Graphs represent mean ± SEM. *P < 0.05, **P < 0.01, 1-way ANOVA followed by Bonferroni’s posttest.

Given the upregulation of LSEC-derived COL4 in liver fibrosis and PHTN, we aimed to investigate the biological function of COL4 in the liver, which is poorly understood. First, we used a conditional LSEC-specific *Col4a1*-mutant mouse in a Cre-dependent manner (37). *Col4a1* mutation was induced by deleting exon 41 (with a floxed DNA sequence under Cre recombinase control) (37) to produce a truncated, dysfunctional COL4 protein (Figure 5A). We detected COL4 expression in liver sinusoids with electronic microscopy via immunogold staining (Figure 5B). In control mice, COL4 was secreted and deposited in the perisinusoidal spaces, whereas *Col4a1* mutation blocked COL4 deposition in liver sinusoids (Figure 5B). This suggests that secreted COL4 is deposited in liver sinusoids and that *Cdh5*^CreERT2^-induced LSEC mutation of *Col4a1* is effective and subsequently decreases sinusoidal COL4 deposition.

Next, we investigated the role of LSEC-derived COL4 in PHTN using LSEC-specific *Col4a1*-mutant mice. After 6-week CCl_4_ administration to induce PHTN, portal pressure was increased 2-fold in the littermate control (*Col4a1*^fl/wt^) mice. In contrast, portal pressure decreased significantly (36%) in the *Col4a1*^fl/wt^ *Cdh5*^CreERT2^ mice compared with *Col4a1*^fl/wt^ mice (Figure 5C). During the development of PHTN, intensified hepatic vascular resistance blocks splanchnic blood flow to liver, which leads to congestion of splanchnic circulation (35). Therefore, we measured spleen weight in control and mutant mice. Indeed, the ratio of spleen weight to body weight was higher in mice that exhibited PHTN by CCl_4_ administration. In contrast, the ratio was decreased by 21% in CCl_4_-treated, *Col4a1*-mutated mice, which was consistent with the change in portal pressure (Figure 5D). Taken together, our data suggest that LSEC-specific COL4 has an important role in PHTN.

Vascular permeability influences blood pressure regulation; decreased vascular permeability increases vascular resistance and thus increases blood pressure (38). To examine whether COL4-induced basement membrane affects sinusoidal permeability, we performed in vivo permeability assays by injecting 4 kDa fluorescein isothiocyanate–dextran (FITC-dextran) via the tail vein in mice administered olive oil (control) or CCl_4_. Sinusoidal permeability was assessed by determining FITC-dextran endocytosed by hepatocytes. The assays showed FITC-dextran at the sinusoidal barrier in the CCl_4_-injected *Col4a1*^fl/wt^ (control) mice (Figure 5E). The amount of FITC-dextran endocytosed by hepatocytes was significantly decreased in CCl_4_-injected *Col4a1*^fl/wt^ mouse liver but was recovered in CCl_4_-injected *Col4a1*^fl/wt^ *Cdh5*^CreERT2^ liver. The data suggest that COL4 restricts sinusoidal permeability because of LSEC capillarization and formation of basement membrane. Thus, LSEC-derived COL4 appears to impair sinusoidal permeability and thus propagates PHTN.

### LSEC-derived COL4 promotes sinusoidal resistance during the progression of PHTN.

**Figure 6:**
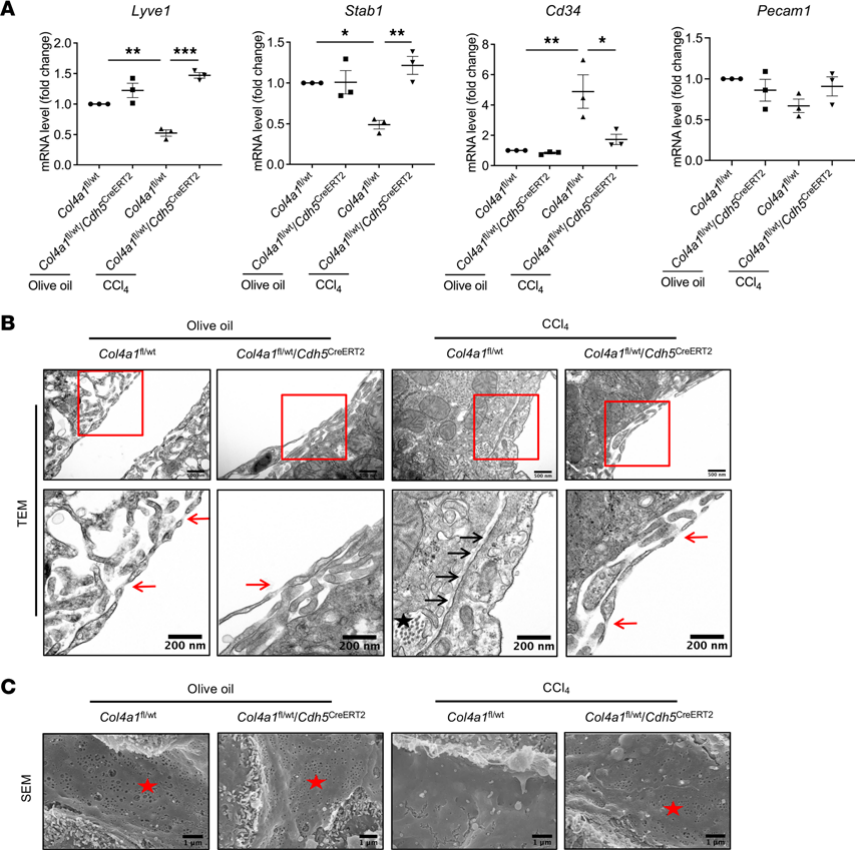
LSEC-specific COL4 promotes sinusoidal resistance. (**A**) After PHTN model establishment, LSECs were isolated for qPCR analysis. Results showed increased expression of *Cd34* (capillarization marker) and decreased expression of *Lyve1* and *Stab1* (LSEC markers) in CCl_4_-induced LSECs. In contrast, *Col4a1* mutation in LSECs maintained the LSEC phenotype, as shown by a decrease in *Cd34* and an increase in *Lyve1* and *Stab1* levels (*n* = 3/group). Graphs represent mean ± SEM. *P < 0.05, **P < 0.01, ***P < 0.001, 1-way ANOVA followed by Bonferroni’s posttest. (**B** and **C**) Healthy LSECs had characteristic fenestrae (red arrows) on transmission electron microscopy (TEM) (**B**) and a sieve plate (red stars) on scanning electron microscopy (SEM) (**C**). Bottom images in **B** are magnifications of red boxes on top images. Scale bars: 500 nm (**B** top), 200 nm (**B** bottom), 1 μm (**C**). In contrast, CCl_4_ induced the defenestration of LSECs, indicated by the loss of fenestrae and formation of basement membrane (black arrows), as well as other collagens’ deposition in perisinusoidal space (black star). *Col4a1* mutation in LSECs restored the LSEC phenotype by maintaining the fenestrae and sieve plate. (*n* = 2/group for TEM and SEM.)

Previous studies have shown CD34 to be a potent marker of LSEC capillarization (39, 40). Given that LSECs are the cellular source of COL4, which has a critical role in PHTN, we hypothesized that COL4 promotes sinusoidal remodeling via induction of sinusoidal resistance. To test that, we first detected capillarization (*Cd34*) and LSEC (*Lyve1*, *Stab1*) markers from isolated mouse LSECs by qPCR. These assays validated that CCl_4_-induced LSECs showed a capillarized phenotype with increased *Cd34* and decreased *Lyve1* and *Stab1* mRNA levels. In contrast, *Col4a1*-mutated LSECs with CCl_4_ administration maintained the LSEC phenotype (Figure 6A). Likewise, CD34 expression was increased and LYVE1 expression was strikingly decreased in human cirrhotic liver as shown by IF staining, which indicates loss of the LSEC phenotype with the development of PHTN (Supplemental Figure 9).

We then directly investigated the ultrastructure of liver sinusoids with electron microscopy. Although the LSECs from healthy livers showed characteristic fenestrae (Figure 6B, red arrow) and the presence of a sieve plate (Figure 6C, red star), LSECs from cirrhotic livers were defenestrated, and the sieve plate was absent (Figure 6, B and C), underneath which obvious basement membrane was observed (Figure 6B, black arrow). Although the perisinusoidal spaces were narrow and without obvious basement membrane deposition, they were widened because of deposition of basement membrane (Figure 6B, black arrow) and other collagens (Figure 6B, black star). In contrast, *Col4a1*-mutant mice with CCl_4_ injection showed an absence of COL4 expression and basement membrane formation in liver sinusoids (Figure 6, B and C). This evidence suggests that COL4 is an important mediator for LSEC capillarization. The remodeled sinusoids contribute to sinusoidal resistance and the development of PHTN.

### COL4 supplies a scaffold for COL1 deposition and assembly in liver sinusoids to promote sinusoidal resistance.

**Figure 7:**
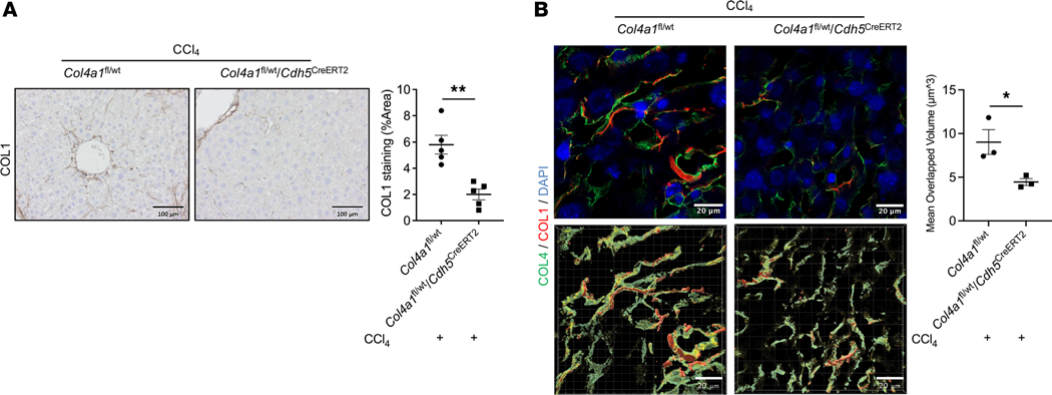
COL4 serves as a scaffold for COL1 deposition and assembly in liver sinusoids to contribute to sinusoidal resistance and stiffness. (**A**) Representative immunohistochemical (IHC) staining of mouse liver tissues showed a significant decrease in COL1 deposition in liver sinusoids from *Col4a1*^fl/wt^ *Cdh5*^CreERT2^ mice compared with *Col4a1*^fl/wt^ mice that had CCl_4_ administration (*n* = 5/group). (**B**) 3D high-resolution images of liver tissues were obtained and reconstructed to show the location of COL1 (red) and COL4 (green) in liver sinusoids (DAPI, blue, indicates the nucleus). The overlapping collagens (yellow) showed that the bulk of the COL1 was surrounded and supported by COL4 in CCl_4_-administered mouse livers. In contrast, COL4 deposition in liver sinusoid was abrogated by *Col4a1* mutation in LSECs. The lack of COL4 scaffold induced patchy and discontinuous COL1 deposition in liver sinusoids (*n* = 3/group). Scale bars: 100 μm (**A**), 20 μm (**B**). Graphs represent mean ± SEM. *P < 0.05, **P < 0.01, 2-tailed unpaired *t* test.

Evidence has shown that COL4, a network-forming ECM, helps form the basement membrane, whereas COL1, a fibril-forming ECM, is secreted by activated HSCs and deposited in the perisinusoidal spaces. By immunohistochemical (IHC) staining of COL1 (Figure 7A), COL1 deposition was evident in CCl_4_-injected mouse liver sinusoids, but the absence of basement membrane formation because of *Col4a1* mutation accompanied decreased COL1 deposition in liver sinusoids. Therefore, we hypothesized that basement membrane supplies a scaffold for COL1 deposition and assembly in the perisinusoidal spaces. To test that, we performed IF staining followed by 3D super-resolution Airyscan (Zeiss) microscopy and reconstructed the images via Imaris Microscopy Image Analysis Software (Oxford Instruments) to illustrate the colocalization of COL4 and COL1 in liver sinusoids. Both COL4 and COL1 were enhanced in liver sinusoids but were distributed differently. A portion of COL1 was structurally surrounded and supported by COL4 via reconstructed images (Figure 7B and Supplemental Video 1). In contrast, sinusoidal COL1 showed a patchy and discontinuous pattern without the structural support of network-forming COL4 in LSEC-specific *Col4a1*-mutated liver sinusoids (Figure 7B and Supplemental Video 2). These findings indicate that COL4 may supply a scaffold for COL1 deposition, which contributes to firm ECM and sinusoidal resistance.

### COL4 contributes to sinusoidal remodeling by activating angiogenic sprouting of LSECs.

**Figure 8:**
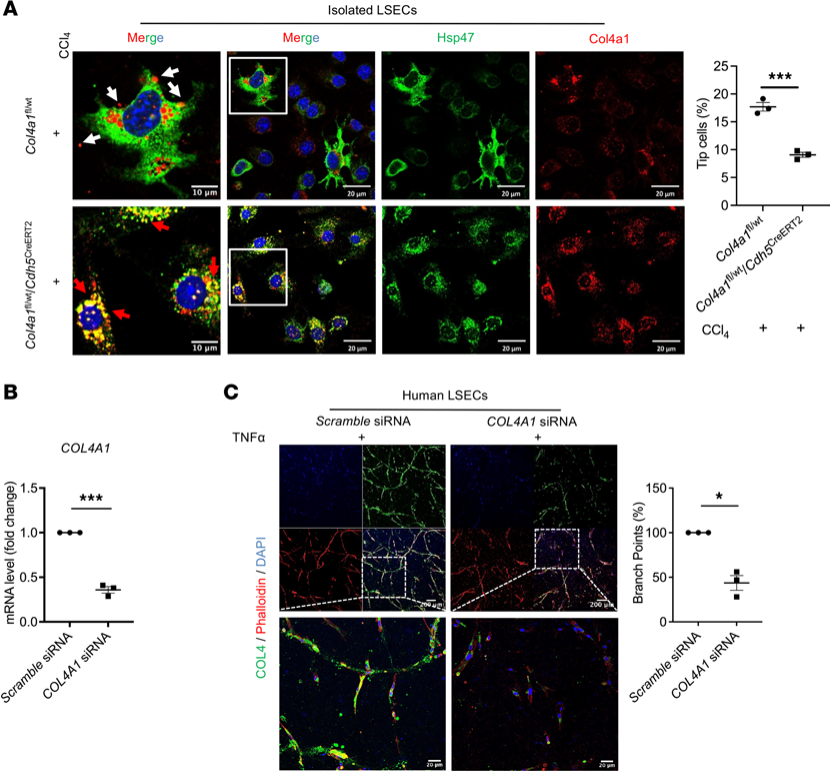
COL4 contributes to sinusoidal remodeling via activating angiogenic sprouting of LSECs. (**A**) Representative IF images of isolated LSECs stained for Col4a1 (red) and Hsp47 (green) (DAPI, blue, indicates nucleus). Mice with PHTN showed Col4a1 production and secretion in LSECs (white arrows), whereas *Col4a1* mutation in LSECs led to dysfunctional Col4a1 retained in endoplasmic reticulum (ER, red arrows). Hsp47 is a chaperone located in ER to help collagen synthesis. Images in the first column are 10 μm magnifications of white boxes in the second column (20 μm, *n* = 3/group). (**B** and **C**) Human LSECs were transfected with *COL4A1* small interfering RNA (siRNA) to knock down the expression of COL4. *Scramble* siRNA was used as a negative control. (**B**) qPCR results showed decreased COL4 expression in human LSECs after *COL4A1* siRNA transfection. (**C**) *COL4A1* siRNA–transfected human LSECs were plated in 3D fibrin gel and treated with TNF-α. Representative IF images of fibrin gel stained for COL4 (green) showed it to be secreted extracellularly and organized into COL4 bundles and tubules. These formed tubes in the *scramble* siRNA group, shown by increased branch points, but *COL4A1* knockdown inhibited tube formation due to the lack of COL4 production and secretion (*n* = 3, biologically independent samples). Green indicates COL4 stained with anti-COL4 antibody; red, phalloidin staining of actin filaments; blue, DAPI staining of DNA. Scale bars: 200 μm (top), 20 μm (bottom). Graphs represent mean ± SEM. *P < 0.05, ***P < 0.001, 2-tailed unpaired *t* test.

Pathologic angiogenesis in fibrotic liver increases intrahepatic blood flow, thus aggravating PHTN (6, 41). To examine the effect of COL4 on angiogenesis, we isolated mouse LSECs from *Col4a1*^fl/wt^ and *Col4a1*^fl/wt^ *Cdh5*^CreERT2^ mice and verified that CCl_4_-injected LSECs transformed into an angiogenic sprouting phenotype, as indicated by filopodial tip cells with abundant COL4 synthesis in the endoplasmic reticulum (ER) and secretion via intracellular vesicles (Figure 8A). In contrast, the *Col4a1* mutation in LSECs inhibited secretion of COL4 and maintained the LSEC phenotype, blocking the activation of LSECs (Figure 8A). These findings suggest that COL4 activates LSEC angiogenic sprouting.

In addition, *Col4a1* mutation led to dysfunctional COL4 being retained in the ER (Figure 8A), which has also been shown previously (37). To further validate that maintenance of the LSEC phenotype is not the result of dysfunctional COL4 retention in the ER, we used an in vitro model to knock down *COL4A1* gene expression in primary human LSECs by small interfering RNA (siRNA) to verify the role of COL4 in angiogenesis. *COL4A1* siRNA showed high efficiency, as reflected by depletion of *COL4A1* mRNA (Figure 8B). We then noted that COL4 was secreted extracellularly and organized into COL4 bundles and tubules in human LSECs transfected with *scramble* siRNA after TNF-α treatment. However, COL4 bundles and tubules were inhibited by *COL4A1* gene knockdown, as shown by decreased branch points (Figure 8C), which suggests that COL4 is essential for LSEC-driven angiogenesis. These results demonstrate that COL4 stimulates LSEC angiogenic sprouting, which increases blood flow in the liver and subsequently propagates the development of PHTN.

### Epigenetic repression of LSEC-derived COL4 alleviates PHTN.

To validate the role of the interaction between specific COL4 enhancer and promoter loci identified in Figure 4 on PHTN, we utilized CRISPRi-dCas9-KRAB epigenome editing in vivo. We used adeno-associated virus (AAV) encoding the sgRNAs sg4 and sg5, which showed high efficacy in targeting the bidirectional promoter, disturbing enhancer activity and silencing *Col4a1* and *Col4a2* expression in cultured primary mouse LSECs (Figure 4A). *dCas9-KRAB Cdh5*^CreERT2^ mice were first injected with tamoxifen to induce LSEC-specific dCas9-KRAB expression before AAV delivery. We then administered the sgRNA AAVs systemically to 8-week-old *dCas9-KRAB Cdh5*^CreERT2^ mice via tail vein injection. LSECs were isolated 2 weeks after AAV-sgRNA delivery, and the repression of *Col4a1* and *Col4a2* mRNA expression was validated (Supplemental Figure 10).

**Figure 9:**
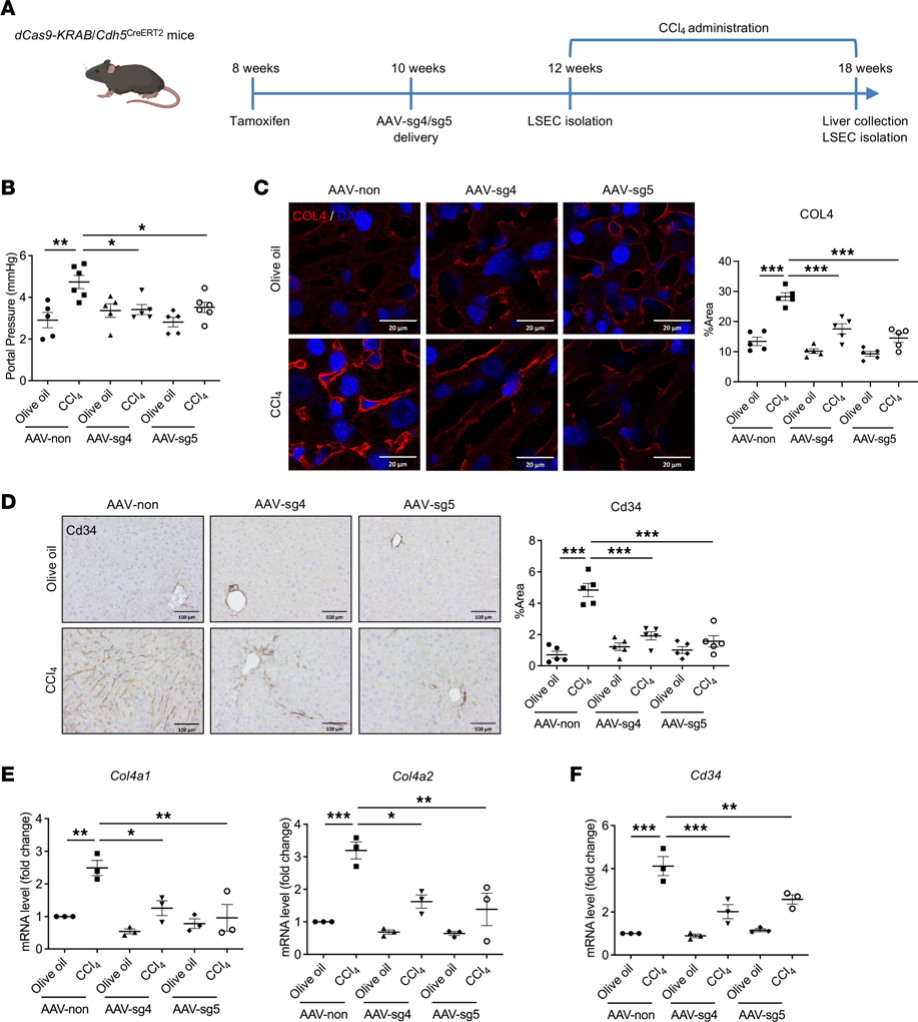
Epigenetic repression of LSEC-derived COL4 alleviates PHTN. (**A**) Schematic of *dCas9-KRAB Cdh5*^CreERT2^ mice undergoing tamoxifen injection, AAV delivery, and PHTN establishment. (**B**) CCl_4_ administration increased portal pressure by 70% in mice with control AAV (AAV-non) delivery, whereas this increase was abrogated by 25% to 30% in mice with AAV-sg4 or AAV-sg5 (*n* = 5–6/group). (**C**) Representative IF staining showed that increased COL4 expression in liver sinusoids after CCl_4_ injection was blocked in mice treated with AAV-sg4 or AAV-sg5 (*n* = 5/group). (**D**) Representative IHC staining of liver tissues showed a significant increase in sinusoidal Cd34 level in mice that underwent CCl_4_ administration and were treated with AAV-non versus with AAV-sg4 or AAV-sg5 (*n* = 5/group). (**E** and **F**) After 6 weeks of CCl_4_ administration, LSECs were isolated for qPCR analysis. Decreased expression of *Col4a1* and *Col4a2* (**E**), as well as decreased expression of *Cd34* (**F**), were noted in mice with AAV-sg4 or AAV-sg5 delivery (*n* = 3/group). Scale bars: 20 μm (**C**), 100 μm (**D**). Graphs represent mean ± SEM. *P < 0.05, **P < 0.01, ***P < 0.001, 1-way ANOVA followed by Bonferroni’s posttest.

We next injected the mice with olive oil or CCl_4_ for establishment of the PHTN model (Figure 9A). CCl_4_ treatment increased portal pressure by 70% in mice administered with the AAV vector containing a nontargeting control sgRNA, which was abrogated when mice were administered AAV-sg4 and AAV-sg5 (Figure 9B).

To validate the role of COL4 in sinusoidal remodeling, we first performed staining showing that sinusoidal COL4 deposition was blocked in mice with administration of AAV-sg4 and AAV-sg5 in addition to CCl_4_ (Figure 9C and Supplemental Figure 11). Consistent with the data, robust Cd34 expression was noted in liver sinusoids in mice with PHTN, which was absent in mice with AAV-sg4 and AAV-sg5 administration (Figure 9D and Supplemental Figure 12). We further verified abrogation of *Col4a1* and *Col4a2* and *Cd34* gene expression in isolated LSECs from mice administered AAV-sg4 and AAV-sg5 (Figure 9, E and F). These results demonstrate high efficiency of AAV delivery and the repressive effect of dCas9-KRAB on the COL4 genes in LSECs in vivo. More importantly, this evidence verifies the pivotal role of COL4 in sinusoidal remodeling and PHTN in vivo.

## Discussion

PHTN is a severe complication of chronic liver disease and a pivotal determinant of disease prognosis and outcomes. Although substantial progress has been made in treating the various causes of chronic liver disease, few therapies are available for the management of PHTN (2, 42). In this study, we provide insights regarding the role of COL4 in the pathogenesis of PHTN. Several observations stand out from our study: 1) COL4 expression is markedly increased in both human and mouse fibrotic livers; 2) LSECs are the cellular source of COL4 in healthy and fibrotic livers, and LSEC-derived COL4 is under the transcriptional regulation of the TNF-α/NF-κB signaling axis; 3) epigenetic repression of the enhancer-promoter interaction silences COL4 gene expression; 4) COL4 induces sinusoidal resistance and angiogenesis, the remodeled sinusoid propagating PHTN; and 5) epigenetic repression of COL4 using CRISPRi-dCas9-KRAB epigenome editing in vivo attenuates PHTN. Taken together, our findings elucidate the mechanism of LSEC-derived COL4 upregulation and the role of COL4 in sinusoidal remodeling and PHTN.

COL4, a major component of basement membrane, has been studied extensively. It has a critical role in different disease states, including intracerebral hemorrhages (37) and glomerular diseases (24, 43), but its role in liver diseases remains elusive. Although a few studies have shown increased COL4 expression in cirrhotic liver (5, 44), the function and cellular source of COL4 in the liver are incompletely explored. Here, we showed that the expression of COL4 increases with the progression of liver cirrhosis and PHTN in human and mouse liver by using an unbiased transcriptome approach in combination with hypothesis-driven studies. Few studies have shown the source of COL4 in the liver (44). By analyzing our scRNA-Seq data and publicly available data sets, we demonstrated that LSECs are the primary source of COL4 and further demonstrated its increase in LSECs from mice with PHTN. Although we showed that HSCs also produce COL4, the expression level is much lower and unchanged between healthy and diseased liver. In contrast, HSCs are the main source of COL1 in cirrhotic liver from our scRNA-Seq data and previous publications (25, 26, 39). Additionally, we conducted a detailed assessment of our scRNA-Seq data of olive oil–injected and CCl_4_-injected mouse livers. Our findings indicated that MMP14, secreted by LSECs, as well as MMP8 and MMP23 from immune cells, displayed no significant alterations in their expression levels. These combined results suggest that the increased deposition of COL4 in both human and mouse fibrotic livers may be the consequence of elevated COL4 expression rather than degradation. In summary, our study may be the first to identify LSECs as the cellular source of COL4 in the liver.

Given the marked increase in COL4 expression in cirrhotic liver and its important role in PHTN, we aimed to identify the signaling pathways leading to this upregulation. TNF-α is the top upstream regulator in the pathogenesis of liver cirrhosis (11) and contributes to loss of the LSEC phenotype (15). However, the underlying mechanism remains elusive. The current study used an LSEC-specific *Col4a1*-mutated and *Col4a1/Col4a2*-repressed model to show that TNF-α/NF-κB signaling leads to LSEC capillarization in a COL4-dependent manner. Nevertheless, the interaction of NF-κB with epigenetic factors to modulate the expression of *Col4a1* and *Col4a2* in LSECs is mechanistically unexplored.

Histone modifications have key roles in enhancer-promoter interactions and are reliable indicators of transcriptional regulatory elements (45). CRISPRi-dCas9-KRAB is a useful approach to epigenetically regulate targeted gene expression with the help of sgRNA (22). We identified decreased enhancer activity when sgRNA targeted the COL4 promoter, as well as decreased promoter activity when sgRNA targeted the COL4 enhancer, shown by the absent enrichment of H3K27ac and H3K4me3 at the corresponding site. Our study indicates that epigenetic repression of the enhancer-promoter interaction silences COL4 gene expression. Given the efficacy and mechanisms of CRISPRi-dCas9-KRAB on COL4 repression, we then used a *dCas9-KRAB Cdh5*^CreERT2^ mouse model together with AAV-sgRNA targeting the *Col4a1/Col4a2* bidirectional promoter to elucidate the function of COL4 in sinusoidal remodeling and PHTN. The data demonstrated that efficient repression of COL4 indeed maintained sinusoidal structure and portal pressure. It is important to note that although CRISPR/Cas9 holds tremendous therapeutic potential, it is still a rapidly evolving field, and ongoing research aims to address safety concerns and improve the precision of the technology.

LSECs maintain sinusoidal communications and homeostasis via angiocrine signaling (46). However, during chronic liver injury, LSECs dedifferentiate to a capillarized phenotype and secrete fibrogenic factors, such as TGF-β and PDGF, which leads to quiescent HSC contraction and activation and subsequently initiates liver cirrhosis and PHTN (5, 47–49). In addition, LSECs induce liver cirrhosis and PHTN by recruiting infiltrated neutrophils in a CXCL1-dependent manner (13) and infiltrated proinflammatory macrophages in a CCL2-dependent manner (12). These inflammatory macrophages produce TGF-β to activate HSCs, as well as TNF-α to defenestrate LSECs, which contributes to liver fibrosis and portal hypertension. All these studies demonstrate the pivotal role of LSECs in the progression of liver fibrosis and PHTN by mediating sinusoidal crosstalk. Whereas sinusoidal crosstalk driven by canonical nitric oxide signaling in portal pressure is well established (47), other mechanisms of LSECs regulating portal pressure are poorly understood. In the current study, we demonstrated that a decrease in functional COL4 production in LSECs lowers the portal pressure in our mouse models of COL4 gene mutation and repression. This suggests a role of LSEC-derived COL4 in PHTN.

The pathobiology of PHTN involves changes in hepatic architecture caused by increased intrahepatic vascular resistance and increased blood flow (42). HSC activation and subsequent ECM deposition distort the liver vascular anatomy and increase liver stiffness, which leads to increased hepatic vascular resistance (25, 50). In this study, we showed that organized, continuous basement membrane formation due to capillarized LSECs increases sinusoidal resistance. Moreover, COL4-enriched basement membrane acts as a scaffold for COL1 deposition in liver sinusoids, which increases sinusoidal stiffness. The absence of COL4 leads to lack of a platform for COL1 deposition in liver sinusoids; unstable fibrillar collagens, such as COL1, without assembly and deposition, might in turn be degraded (51). The glycoprotein fibronectin binds and interacts with collagen and is involved in collagen assembly and deposition (52). A previous publication showed spatial accumulation of fibronectin adjacent to the basement membrane (53). Currently, the relationship between COL4 and COL1 is largely unknown, and fibronectin is speculated to be a mediator linking COL1 on a COL4 scaffold, but this needs further investigation. Collectively, our data indicate that LSEC-derived COL4 mediates LSEC capillarization and basement membrane formation, contributing to sinusoidal resistance during the development of PHTN.

Besides increased sinusoidal resistance, angiogenesis-induced blood flow in the liver contributes to PHTN. Intrahepatic angiogenesis occurring after liver injury drives pathologic sinusoidal remodeling and increases the vascular volume, further propagating PHTN (16, 54, 55). Studies have demonstrated that inhibition of vascular endothelial growth factor is beneficial for attenuating intrahepatic vascular remodeling and PHTN (56). Our study indicates that COL4 is an important factor mediating angiogenesis, as shown by angiogenic sprouting of mouse and primary human LSECs. COL4 has been shown to be involved in pathological retina angiogenic sprouting (57) and lung endothelial cell angiogenesis in vivo (58). In that work, the authors observed the number of filopodia at the angiogenic front was decreased along with the reduction of functional secreted COL4 (57) and further verified that COL4 contributed to tube formation of lung endothelial cells through integrin/FAK signaling by knocked down expression of COL4 (58). Taken together, our data show that COL4 directly affects portal pressure by sinusoidal resistance and angiogenesis. In conclusion, our work implicates the mechanisms of LSEC-derived COL4 regulation and its role in the pathophysiology of PHTN. COL4 can potentially become a therapeutic target in PHTN.

## Methods

### Sex as a biological variable

In this study, we included both female and male individuals. The results showed consistency across sexes, and no distinctions were made based on sex in the analysis or interpretation of the findings.

### In vivo experiments

*Col4a1*^fl/wt^ mice were provided by Douglas B. Gould at the University of California, San Francisco, San Francisco, California, USA. *Col4a1*^fl/wt^ mice were crossed with *Cdh5*^CreERT2^ mice to generate *Col4a1*^fl/wt^ *Cdh5*^CreERT2^ mice. Mice at 6 to 7 weeks old were intraperitoneally injected with tamoxifen (75 mg/kg/d) for 5 consecutive days to induce LSEC-specific *Col4a1* mutation. To induce liver fibrosis–related PHTN, CCl_4_ (MilliporeSigma 319961) at 1 μL/g of body weight was intraperitoneally administered twice per week for 6 weeks. Mice were humanely killed 48 hours after the last injection.

*dCas9-KRAB* mice were purchased from The Jackson Laboratory (033066). *dCas9-KRAB* mice were crossed with *Cdh5*^CreERT2^ mice to obtain *dCas9-KRAB Cdh5*^CreERT2^ mice. Tamoxifen was administered to induce dCas9-KRAB expression in LSECs. sgRNA targeting the bidirectional promoter or enhancer region for the *Col4a1* and *Col4a2* genes was delivered into *dCas9-KRAB Cdh5*^CreERT2^ mice via the tail vein before 6-week CCl_4_ injections.

### Portal pressure measurement

The mouse portal vein was cannulated with a 24-gauge catheter attached to a pressure transducer and connected to a Digi-Med Blood Pressure Analyzer (BPA-400) to measure portal pressure.

### Liver sinusoid permeability assay

Mice were anesthetized with isoflurane. Permeability of the liver sinusoids was measured by FITC-dextran injection. Briefly, 200 μL of a 0.01 mg/mL 4-kDa FITC-dextran solution in saline was injected via the tail vein. Five minutes later, mice were killed, and the liver was harvested, embedded in optimal cutting temperature compound (Tissue-Tek O.C.T. Compound; Sakura 4583), and flash-frozen on dry ice. The frozen liver was cut into 7 μm sections on a cryostat (Leica Microsystems) and mounted on glass slides. Liver sinusoid permeability was analyzed by determining the amount of FITC-dextran endocytosed by hepatocytes with ImageJ software (NIH).

### Mouse LSEC isolation and culture

Mouse LSECs were isolated from *Col4a1*^fl/wt^, *Col4a1*^fl/wt^ *Cdh5*^CreERT2^, and *dCas9-KRAB Cdh5*^CreERT2^ mice as previously described (59). Briefly, mice were anesthetized with isoflurane before the liver was perfused with phosphate-buffered saline (PBS) containing proteases (Roche 25551121) and collagenase P (Roche 11249002001). The perfused liver was homogenized and filtered through a 70 μm cell strainer (Falcon 352350) to obtain a single-cell suspension. Anti-CD146 microbeads (Miltenyi Biotec 130-092-007) then were used to purify LSECs according to the manufacturer’s protocols. The purified LSECs were plated on a COL1-coated dish and glass chamber and cultured in Endothelial Cell Medium (ScienCell Research Laboratories 1001) for 4 hours. The cells were harvested for further analysis.

### Cell treatment and siRNA transfection

Human LSECs (ScienCell Research Laboratories 5000) were cultured according to manufacturer instructions. To study COL4 expression, low-passage cells were starved with low-serum medium (0.5% fetal bovine serum [FBS] in basal endothelial medium) for 2 hours followed by treatment with NF-κB inhibitor (celastrol, 2 μM; MilliporeSigma C0869) for 2 hours. Next, human recombinant TNF-α (20 ng/mL; PeproTech, 300-01A) was added to low-serum medium and incubated with cells for 4 to 24 hours before cells were collected for further analysis (qPCR and IF staining). For siRNA transfection, human LSECs at 70% confluence were transfected with *COL4A1* or *scramble* siRNAs (ON-TARGETplus SMARTpool; Dharmacon, Horizon Discovery) using the Lipofectamine RNAiMAX Transfection Reagent (Thermo Fisher Scientific) according to manufacturer instructions. Cells were collected after 48 hours of transfection.

### TAF–ChIP-Seq analysis

TAF–ChIP-Seq was performed at the Epigenomics Development Lab at Mayo Clinic by using antibodies against histone mark H3K27ac (Cell Signaling Technology 8173). Briefly, mouse LSECs were isolated and cross-linked with 1% formaldehyde for 10 minutes, followed by quenching with 125 mM glycine for 5 minutes at room temperature and by washing with PBS. The pellets were resuspended in lysis buffer and incubated on ice before sending them to the epigenomics lab.

### RNA-Seq and analysis

RNA-Seq was performed on whole liver from human samples of healthy and alcohol-induced cirrhotic liver at the Mayo Clinic Center for Individualized Medicine Medical Genome Facility. Some of the raw RNA-Seq data have been previously published by our group and are available on the National Center for Biotechnology (NCBI) Gene Expression Omnibus (GEO) database (GSE155907). The detailed protocols were described in a previous paper (14).

### scRNA-Seq and analysis

The scRNA-Seq analysis depicted in Figure 2B was based on a pooling strategy that combined purified hepatocytes (~1/3 of the entire cell population), enriched HSCs (~1/3), and other nonparenchymal cells (~1/3), which encompassed cell types like LSECs, Kupffer cells, and others. It is important to note that this pooling strategy does not reflect the actual cell number ratios, particularly concerning LSECs and HSCs, in vivo and rather results in an overrepresentation of HSCs. HSC isolation has been described previously (27). Mouse hepatocytes and liver nonparenchymal cells were isolated after the mice underwent 6 weeks of administration of olive oil or CCl_4_. Briefly, livers were perfused with PBS containing proteases and collagenase P before they were homogenized and filtered through a 70 μm cell strainer to obtain a single-cell suspension. The cell suspension was centrifuged at 50*g* for 3 minutes at 4°C to remove hepatocytes and saved for sequencing. The supernatants were pelleted at 500*g* for 5 minutes at 4°C and resuspended in Endothelial Cell Medium. Samples were then prepared according to the Chromium Single Cell 3′ v2 Reagent Kit (10x Genomics) user guide. Briefly, liver nonparenchymal cells were pelleted and resuspended in PBS + 0.04% bovine serum albumin to 1,000 cells/μL. Cell viability was evaluated with trypan blue, and cell number was verified with an automated cell counter. Samples were loaded onto the Chromium Single Cell-A chip (10x Genomics) to convert polyadenylated mRNA into cDNA. cDNA was amplified by PCR for library generation. Samples were sequenced on a HiSeq 2500 system (Illumina) at the Mayo Clinic Center for Individualized Medicine Medical Genome Facility with the following run parameters: read 1, 26 cycles; read 2, 98 cycles; index 1, 8 cycles. scRNA-Seq data sets were annotated by CellMarker 2.0 databases (https://doi.org/10.1093/nar/gkac947).

### qPCR

RNA extraction from cells and liver tissues was performed with the RNeasy Mini Kit (QIAGEN 74104) according to the manufacturer’s protocols. RNA 500 ng was used for cDNA synthesis with oligo primer and deoxynucleotide triphosphates using SuperScript III First-Strand Synthesis System (Invitrogen 18080-051) after RNA quantification by spectrophotometry (NanoDrop, Thermo Fisher Scientific). qPCR was performed using iTaq Universal SYBR Green Supermix (Bio-Rad 1725120) according to the manufacturer’s instructions. For murine samples, mRNA levels were normalized to the housekeeping gene β-actin. For human samples, mRNA levels were normalized to the housekeeping gene GAPDH. Primer sequences are listed in Supplemental Table 1.

### Western blot assay

Cells or liver tissues were lysed in RIPA buffer (Cell Signaling Technology 9806S) with Complete, Mini, EDTA-free protease inhibitor cocktail (Roche 4693159001). Equal amounts of protein for cell lysates (15 μg) or liver lysates (30 μg) were loaded onto SDS-PAGE and transferred to a nitrocellulose membrane. The membrane was incubated overnight with primary antibodies after blocking with 5% blotting-grade blocker (Bio-Rad 1706404) for 1 hour. Primary antibodies were used to detect HSC70 (Santa Cruz Biotechnology sc-7298) and CAS9 (EnCor Biotechnology MCA-3F9). The signal of the blot was developed and detected using chemiluminescence substrate (Western Blotting Luminol Reagent, Santa Cruz Biotechnology sc-2048, or Immobilon Crescendo Western HRP substrate, MilliporeSigma WBLUR0100). HSC70 was used as the loading control, and the results were quantified with ImageJ software.

### IHC staining

Mouse livers were fixed in 10% formalin, embedded in paraffin, and cut into 5 μm sections. Slides were deparaffinized, and antigen retrieval was performed (IHC-Tek; IHC WORLD IW-1000) before blocking in 10% FBS for 1 hour at room temperature and incubating overnight at 4°C with primary antibodies: anti-COL1 (Southern Biotech 1310-01) or anti-CD34 (Abcam ab81289). After incubation with biotinylated secondary antibody (Vector Laboratories BA-1100) for 1 hour at room temperature, slides were treated with VECTASTAIN Elite ABC-HRP Reagent, Peroxidase (Vector Laboratories PK-7100) for 30 minutes before treating with DAB Substrate Kit, Peroxidase (Vector Laboratories SK-4100), for 3 minutes. Slides were counterstained with hematoxylin and dehydrated in ethanol and xylene. Images were acquired on a histologic microscope (ZEISS) at ×20 and ×40 original magnification, with 6 images per slide.

### IF staining

Frozen liver tissues were cut into 7 μm sections and fixed in acetone for 10 minutes and permeabilized with 0.5% Triton X-100. Slides were blocked in 10% FBS for 1 hour at room temperature before being incubated with primary antibody overnight at 4°C. Primary antibodies and stains used included anti-COL4 (Southern Biotech 1340-01; Abcam ab19808), anti-COL1 (Southern Biotech 1310-01), anti-CD34 (Abcam ab81289), anti-LYVE1 (R&D Systems AF2125), Phalloidin-TRITC (MilliporeSigma P1951), anti-COL4a1 (Chondrex 7070), anti-COL4 (Abcam ab236640), anti-PDGFRβ (Cell Signaling Technology 3169), anti–α-SMA (Abcam ab7817), and anti-Hsp47 (Novus Biologicals NBP1-97491). After incubation with fluorochrome-coupled secondary antibodies (Invitrogen, A11055, A11057, A21206, A11077, A21202, A10042) and DAPI, images were visualized on a Zeiss LSM 780 confocal microscope. 3D super-resolution images were visualized on a Zeiss LSM 980 Airyscan microscope and reconstructed and viewed with Imaris image analysis software. Colocalization analysis was done as described previously (60). Three sinusoidal areas were selected for analysis. Fluorescence intensity peaks and ratio of green fluorescence versus red fluorescence were quantified by Zen Image Browser at 4 straight arrows as shown in the schema of hepatic sinusoids (Supplemental Figure 2D).

### sgRNA design and transfection

sgRNA sequences targeting the bidirectional promoter or putative enhancer region of *Col4a1/Col4a2* were designed using public Benchling software and synthesized by Horizon Discovery. For isolated LSECs from *dCas9-KRAB Cdh5*^CreERT2^ mice, synthetic sgRNA was transfected using DharmaFECT Duo Transfection Reagent (Horizon Discovery) according to the manufacturer’s protocols for 18 hours before mouse recombinant TNF-α was administered for 24 hours. The cells then were harvested for qPCR or ChIP-qPCR analysis. The sgRNA sequences are listed in Supplemental Table 2.

### AAV subcloning, production, and delivery

sg4 and sg5, which revealed high efficiency in silencing COL4 gene expression, were selected to subclone into the AAV backbone. An AAV backbone encoding sgRNA (pAAV-U6-sgRNA-CMV-GFP) was obtained from Vector Biolabs. An AAV encoding LacZ (pAAV-U6-LacZ-CMV-GFP) was used as the nontargeting control. High-titer AAVs for systemic delivery were produced in serotype AAV2 by Vector Biolabs. *dCas9-KRAB Cdh5*^CreERT2^ mice were injected with AAV-sg4 (1 × 10^11^ viral genomes/mouse), AAV-sg5 (1 × 10^11^ viral genomes/mouse), or AAV-non (1 × 10^11^ viral genomes/mouse) via the tail vein using a 31-gauge needle. LSECs were isolated to detect Col4a1 and Col4a2 expression 2 weeks after AAV delivery.

### Electron microscopy

Anesthetized mice were perfused with Trump’s fixative (Electron Microscopy Sciences 11750) via the portal vein. Dissected liver lobes were subsequently immersed in freshly prepared aldehyde fixative overnight before sending these samples to the Microscopy and Cell Analysis Core at Mayo Clinic for further processing.

#### Transmission electron microscopy.

Tissue was placed into fixative (4% paraformaldehyde + 1% glutaraldehyde in 0.1 M PBS, pH 7.2). After fixation, tissue was washed with PBS, stained with 1% osmium tetroxide, washed in distilled water, stained with 2% uranyl acetate, washed in distilled water, dehydrated through a graded series of ethanol and acetone, and embedded in EMbed 812 resin (Electron Microscopy Sciences 14120). After 24 hours of polymerization at 60°C, 0.1 μM ultrathin sections were prepared and poststained with lead citrate. Micrographs were acquired at 80 kV using a JEM-1400 Plus transmission electron microscope (JEOL, Inc) equipped with a Gatan Orius camera (Gatan, Inc).

#### Scanning electron microscopy.

After fixing in Trump’s fixative at 4°C overnight, livers were washed in PBS, rinsed in water, and dehydrated through a graded series of ethanol. It was then critical point–dried using carbon dioxide, mounted on an aluminum stub, and sputter-coated with gold-palladium for 90 seconds. Finally, livers were imaged in a Hitachi S-4700 cold field emission scanning electron microscope at 5 kV accelerating voltage.

### Immunogold staining

Livers were first perfused with 0.1% glutaraldehyde and 4% paraformaldehyde in 0.1 M PBS for 2 hours. The sample was then cryoprotected by immersion in 2.3 M sucrose in 0.1 M PBS overnight and frozen in liquid nitrogen. Cryosections 5 to 6 μm thick were cut with a cryomicrotome (Leica Microsystems). Samples were incubated overnight at 4°C with anti-COL4 antibody (Abcam, ab19808) diluted 1:20 in 10% fetal calf serum/PBS. Sections were then incubated with goat anti-rabbit Ultra Small ImmunoGold secondary antibody (Electron Microscopy Sciences 25101) for 2 hours at room temperature. Sections were further fixed in 1% glutaraldehyde for 15 minutes; then silver enhancement was performed with R-Gent Silver Enhancement Reagents, SE-EM (Electron Microscopy Sciences), for 30 minutes and postfixed with 1% osmium tetroxide for 30 minutes. After washing with distilled water, the sections were dehydrated with serial alcohol, infiltrated, and embedded with Spurr resin. The specimens were cut at 90 nm thickness and stained with lead acetate, then viewed at 80 kV using a JEM-1400 Plus electron microscope.

### ChIP-PCR

Mouse LSECs were isolated according to the previous protocol. LSECs were transfected with sgRNA and underwent ChIP with the Magna ChIP HiSens Chromatin IP Kit (MilliporeSigma MAGNA0025) according to the manufacturer’s protocol. Briefly, cells were cross-linked, washed, collected, and lysed with lysis buffer. The nucleus was extracted using nuclear lysis buffer. Chromatin (10 μg per IP reaction) was sonicated, centrifuged at 10,000*g* at 4°C for 10 minutes, and immunoprecipitated with magnetic beads with antibodies against H3K9me3 (Abcam ab8898), H3K27ac (Abcam ab4729), and H3K4me3 (Diagenode C15410003). Isotype IgG (MilliporeSigma 12-370) was used as control.

### Statistics

All data were analyzed with GraphPad Prism software (version 9.0). Data represent mean ± standard error of the mean and were analyzed by 1-way ANOVA or a 2-tailed *t* test with post hoc Bonferroni’s correction. *P* < 0.05 was considered statistically significant.

### Study approval

Human liver samples were collected at Mayo Clinic with institutional approval (IRB 15-008251) after receipt of informed consent from patients. All animal experiments were conducted in accordance with guidelines approved by the Mayo Clinic Institutional Animal Care and Use Committee.

### Data availability

All relevant data supporting the findings of this study are reported within the article or its supplemental material, including the Supporting Data Values file. The scRNA-Seq data generated in this publication are available on the GEO database (GSE199064). The TAF-ChIP-Seq data generated in this publication are available on the GEO database (GSE233284). The RNA-Seq data are available on the GEO database (GSE155907). The ChIP-Seq data are available on the GEO database (GSE155908, GSE31039, GSE53998).

## Author contributions

CG contributed to study design, animal and cell experiments, data acquisition and analysis, and drafting of the manuscript. UY, MX, AA, and NJS contributed to genotyping and animal experiments. JL and SJ contributed to cell experiments. ANC and EK contributed to CRISPR and epigenetic studies. TSS contributed to genomic sequencing studies and associated data analysis. NWH contributed to revision of the manuscript. SC contributed to study design, data analysis, intellectual input, and revision of the manuscript. VHS contributed to study design, resources, funding support, revision of the manuscript, and overall study supervision.

## Supplementary Material

Supplemental data

Supplemental video 1

Supplemental video 2

Supporting data values

## Version 1 05/07/2024

In-Press Preview

## Version 2 06/10/2024

Electronic publication
