# Supplementary material for: Liver sinusoidal endothelial cells contribute to portal hypertension through collagen type IV–driven sinusoidal remodeling
Source: JCI Insight. 2024 May 7;9(11):e174775. doi: 10.1172/jci.insight.174775 (PMC11382879; doi:10.1172/jci.insight.174775)

Supplementary Table 1. Primer Sequences

Primer name	Sequence
qPCR primers	
Human *COL4A1* Forward	CCAGGGGTCGGAGAGAAAG
Human *COL4A1* Reverse	GGTCCTGTGCCTATAACAATTCC
Human *COL4A2* Forward	ACAGCAAGGCAACAGAGG
Human *COL4A2* Reverse	ACAGCAAGGCAACAGAGG
Human *CXCL1* Forward	AACCGAAGTCATAGCCACA
Human *CXCL1* Reverse	CCTCCCTTCTGGTCAGTTG
Human *GAPDH* Forward	CCAGGGCTGCTTTTAACTCT
Human *GAPDH* Forward	GGACTCCACGACGTACTCA
Mouse *Col4a1* Forward	CTGGCACAAAAGGGACGAG
Mouse *Col4a1* Reverse	ACGTGGCCGAGAATTTCACC
Mouse *Col4a2* Forward	CCCGGATCTGTACAAGGGTG
Mouse *Col4a2* Reverse	TGATGCCTTCCTCGCCTTTT
Mouse *Lyve1* Forward	TACAGGACCCATGGCTGAGA
Mouse *Lyve1* Reverse	GGTGCCAAGCATTTCGGTTT
Mouse *Cd34* Forward	CAGGAGAAAGGCTGGGTGAAG
Mouse *Cd34* Reverse	GTTGTCTTGCTGAATGGCCG
Mouse *Pecam1* Forward	TGGTTGTCATTGGAGTGGTC
Mouse *Pecam1* Reverse	TTCTCGCTGTTGGAGTTCAG
Mouse *Stab1* Forward	CATTGTGCAACGGCACTTGA
Mouse *Stab1* Reverse	CCCACAGATGCTGCAAGTCT
Mouse *β-actin* Forward	TGACGTTGACATCCGTAAAG
Mouse *β-actin* Reverse	GAGGAGCAATGATCTTGATCT
ChIP H3K4me3 Promoter Forward	GATCGGAGCTGGGGGAATAG
ChIP H3K4me3 Promoter Reverse	CTCCGCATAATGTCCCTCGT
ChIP H3K27ac Enhancer Forward	GAGCTGTAGGTCAGTGGTGC
ChIP H3K27ac Enhancer Reverse	ATGGAACCCAGGCTCTCCT
ChIP H3K9me3 Promoter Forward	AGCTGGGGGAATAGGGATCG
ChIP H3K9me3 Promoter Reverse	GCACCCTGACTCCGCATAAT
ChIP H3K9me3 Enhancer Forward	TGGTGCAGCACTTGCCTA
ChIP H3K9me3 Enhancer Reverse	GCCTGGCATGGAACCCA
Primers for genotyping	
Mouse *Cdh5-CreERT2* Forward	GCCTGCATTACCGGTCGATGCAACGAGTG
Mouse *Cdh5-CreERT2* Reverse	CTGGCAATTTCGGCTATACGTAACAGGGTG
Mouse *Col4a1* Forward	TGATAGAAGAGCTGTCGTGGGAG
Mouse *Col4a1* Reverse	CTTCATCCCGTCGAGTGGGTGG
Mouse *dCas9-KRAB* Forward 1	GCAGCCTCTGTTCCACATACAC
Mouse *dCas9-KRAB* Reverse	TAAGCCTGCCCAGAAGACTC
Mouse *dCas9-KRAB* Forward 2	AAAGTCGCTCTGAGTTGTTAT

Supplementary Table 2. Panel of sgRNA protospacer sequences targeting promoter and enhancer region of *Col4a1*/*Col4a2*

sgRNA #	Protospacer	PAM	Strand
1	CTGCGACACCAAGTCCCGAG	CGG	-
2	GTCACCCCGCAAGGAAACGA	GGG	+
3	TCGATCCCTTCCTAACCTGG	AGG	-
4	AAATTGGGGAGCTCCTCGGG	CGG	-
5	AGGTGAGAGCGACCCCCGGG	AGG	+
6	TGCGCAGAGTCACCGCACCC	GGG	-
7	CGCAGGGAGTCCGAACGCCG	GGG	+
8	ACGGGCCAACGCTTCTTCAG	GGG	+
9	GTTGGCCCGTGGCACACCGG	CGG	-
10	CGAGACTGAGCACCTCGCCG	TGG	-
EL1_1	AGGTTCTACGTGATCTATGG	AGG	-
EL1_2	CAACACGTCCTTGAGTCTGG	GGG	-
EL1_3	AACACTCCCAACACGCAGCA	GGG	+
EL2_1	ATATCACCTGATGACCCTGG	TGG	+
EL2_2	TGGAGAGAAGGGTAGCATCG	GGG	-
EL2_3	GCCCTGGGACTATTGGTACA	AGG	+
EL2_4	AGGGGACACCTGGAGTTCCA	GGG	-
ER1_1	TGTTGTTCTAGCTACTTGAG	GGG	+
ER1_2	CAGGATCTACCAGATCATCC	AGG	-
ER1_3	AATCCAAAAATTCCTGACTG	AGG	+
ER2_1	GCTACGCAAAGCCTCCCTTG	TGG	-
ER2_2	CTGAGGTCCCAGAATAGCCG	AGG	-
ER2_3	ACACAATCACTATGGGTCCT	GGG	+
ER3_1	GGGAAAGCAAGAATCCAGGT	TGG	+
ER3_2	CCTGTGTGGTTGAGTGTCCG	TGG	+
ER3_3	ACTGGACATGTGACCGACAT	TGG	+

Supplementary Figure Legends

Supplementary figure 1. RNA-Seq analysis of human healthy and cirrhotic livers reveal differentially expressed genes. Bulk RNA-Seq analysis (GSE155907) was performed on human healthy, and alcohol (AH)-induced cirrhotic livers and identified 950 upregulated genes and 761 downregulated genes in human cirrhotic livers.

Supplementary figure 2. HSCs are the main source of *Col1a1* and *Col3a1*. Single-cell RNA-Seq (scRNA-Seq) analysis was performed on normal (olive oil) and fibrotic (CCl_4) mouse livers subjected to olive oil and CCl_4 administration, respectively. (**A**) tSNE analysis identified 17 main cell clusters (numbered) in the liver using distinct gene markers. Among them, *Pdgfra* and *Pdgfrb* were marked for HSCs, which represented Cluster 5. Each orange dot represents an individual HSC. (**B**) Violin plots for scRNA-seq data from the 17 cell clusters revealed that *Col1a1* and *Col3a1* were mainly expressed by HSCs from fibrotic livers. (**C**) By analyzing percentage of cells expressing the gene, log2FC of gene in fibrotic vs healthy livers, and adjusted $P<.05$ (Adjusted P-value from DESeq2), the 3D plot showed increased *Col1a1* and *Col3a1* in HSC cluster in mouse fibrotic livers. (D) Illustration of the liver sinusoid with four arrows is presented to facilitate the analysis of colocalization between markers for Liver Sinusoidal Endothelial Cells (LSECs) and Hepatic Stellate Cells (HSCs) with Collagen IV (COL4). The immunostaining of the liver sinusoid was examined for the colocalization of COL4 with LYVE1 (LSEC marker), PDGFRβ, and αSMA at four distinct levels indicated by red arrows. The image intensity was quantified using Zen image browser. Representative Immunofluorescence (IF) images display COL4 staining in green and αSMA staining in red. Adjacent to each image, colocalization analyses are presented for each merged image. The graphs depict the average fluorescence

intensities of the staining at specified distances (in nanometers) along the four straight white arrows in each sinusoid. The white arrows in the merged images indicate the selected areas for colocalization. Each graph enumerates the intensity peaks for each fluorophore (green and red). The ratio of intensity peaks of COL4 (green) vs αSMA (red) is indicated at the top of graph (E). Similarly, the ratio of intensity peaks of COL4 (red) vs PDGFRβ (green) staining is also depicted (F). Colocalization analysis was performed on three complete sinusoids (n=3/group). DAPI (blue) was employed for nuclei staining. Notably, IF images reveal the absence of colocalization between COL4 and αSMA (E) and PDGFRβ (F).

Supplementary figure 3. Publicly accessible databases show source and expression level of COL4 in normal and diseased livers from human and mouse samples. (**A**) FANTOM5 human liver RNA-Seq data revealed LSECs produced more *COL4A1* and *COL4A2* while HSCs produced more *COL1A1* and *COL3A1* (n=3, biologically independent samples). (**B**-**C**) t-SNE analysis using public Tabula Muris data sets revealed that *Col4a1* (**B**) and *Col4a2* (**C**) were mainly expressed by LSECs in mouse livers. (**D**) The increase in *Col4a1* and *Col4a2* expression in LSECs from mouse NASH livers by analyzing published databases (n=3, biologically independent samples). Graphs represent mean ± SEM. **P*<.05, 2-tailed unpaired Student's *t* test.

Supplementary figure 4. TNF**α** and NF**κ**B are top upstream regulators in human cirrhotic livers. IPA analysis demonstrated that TNFα and NF-κB were the top upstream regulators of differentially expressed genes by analyzing bulk RNA-Seq from our human cirrhotic livers (GSE155907).

Supplementary figure 5. NF-**κ**B enrichment at bi-directional promoter and putative enhancer regions of endothelial COL4 genes in response to TNF**α** stimulation. ChIP-Seq data sets from GEO public database (GSE53998) revealed that occupancy of NF-κB at the bi-directional promoter region (black rectangle), as well as 5 putative enhancer regions (red rectangles) of COL4 genes after TNFα stimulated HUVEC.

Supplementary figure 6. Histone enrichment at bi-directional promoter and putative enhancer regions of COL4 genes in mouse liver. (**A**) To identify the putative enhancer regions of COL4 genes in mouse LSECs, we performed TAF-ChIP-Seq analysis on isolated mouse LSEC and noted occupancy of H3K27ac at the bi-directional promoter region (black rectangle) and 5 putative enhancer regions (red rectangles) of COL4 genes. (**B**) ChIP-Seq data sets from GEO public database (GSE31039) showed that comparable occupancy of H3K27ac at the bi-directional promoter region (black rectangle) and putative enhancer regions (red rectangles), occupancy of H3K4me3 at the bi-directional promoter region (black rectangle), and occupancy of H3K4me1 at the enhancer regions (red rectangles) of COL4 genes in mouse liver.

Supplementary figure 7. Histone enrichment at bi-directional promoter and putative enhancer regions of human COL4 genes in alcohol-induced human cirrhotic liver. ChIP-Seq data sets from GEO public database (GSE155908) revealed increased occupancy of H3K27ac at the bi-directional promoter region (black rectangle) and putative enhancer regions (red rectangles), increased occupancy of H3K4me3 at the bi-directional promoter region (black rectangle) of COL4 genes in human cirrhotic liver.

Supplementary figure 8. Schematic of sgRNA targeting sites at the bi-directional promoter and enhancer regions of COL4 genes in mouse LSEC. Isolated LSECs from *dCas9-KRAB/Cdh5*$^{\mathrm{CreERT2}}$ mice after in vivo tamoxifen injection were transfected with sgRNAs, which target the bi-directional promoter region or putative enhancer regions of the COL4 genes. Non-targeting sgRNA was used as a negative control. (**A**) sgRNAs targeting different loci of *Col4a1/Col4a2* were designed via Benchling software: 10 sgRNAs (black arrows) targeting the bi-directional promoter region and 16 sgRNAs (red arrows) targeting the putative enhancer regions. (**B**) Western blot assay was performed on LSECs from *dCas9-KRAB/Cdh5*$^{\mathrm{CreERT2}}$ mice subjected to tamoxifen injection. dCas9-KRAB protein in LSECs was induced by tamoxifen. HSC70 was used as loading control. (**C-D**) sgRNAs targeting COL4 promoter region (**C**) and putative enhancer regions (**D**) were transfected to the isolated LSECs from *dCas9-KRAB/Cdh5*$^{\mathrm{CreERT2}}$ mice expressing dCas9-KRAB. *Pecam1* gene expression in LSECs showed no differences among groups via qPCR assay (n=3-4, biologically independent samples).

Supplementary figure 9. LSEC phenotype is damaged in human cirrhotic livers. Representative IF images showed increased CD34 (red) expression but decreased LYVE1 expression (green) in human cirrhotic livers as compared to healthy livers. DAPI (blue) was used to stain nuclei (n=3/group). Graphs represent mean ± SEM. **P*<.05, ***P*<.01, 2-tailed unpaired Student's *t* test.

Supplementary figure 10. *Col4a1* and *Col4a2* were repressed in mouse LSECs after AAV-sg4/sg5 delivery to *dCas9-KRAB/Cdh5*$^{\mathrm{CreERT2}}$ mice. (**A**-**B**) One week after tamoxifen injection, *dCas9-KRAB/Cdh5*$^{\mathrm{CreERT2}}$ mice were delivered by AAV encoding sg4 or sg5, both of which target COL4 promoter to repress *Col4a1* and

Col4a2 gene expression. AAV encoding LacZ is regarded as a negative control (AAV-non). Two weeks after AAV delivery, LSECs were isolated and showed significant decrease in *Col4a1* and *Col4a2* gene expression in AAV-sg4 (**A**) and AAV-sg5 (**B**) groups (n=3/group). Graphs represent mean ± SEM. *$P<.05$, **$P<.01$, 2-tailed unpaired Student's t test.

Supplementary figure 11. Decrease in liver COL4 expression after AAV-sg4/sg5 were delivered to mouse with PHTN. Representative IF staining was performed on liver tissues and showed that increased COL4 expression in liver sinusoid after CCl_4 treatment was blocked in mice with AAV-sg4 or AAV-sg5 delivery. Red indicates COL4 stained with anti-COL4 antibody; blue, DAPI staining of DNA. Images from Figure 9C are magnifications of white boxes on the images.

Supplementary figure 12. Decrease in liver CD34 expression after AAV-sg4/sg5 were delivered to mouse with PHTN. Representative IHC staining was performed on liver tissues and exhibited the significant increase in sinusoidal Cd34 level from mouse treated with control AAV compared to mice with AAV-sg4 or AAV-sg5 underwent CCl_4 administration. Images from Figure 9D are magnifications of black boxes on the images.

Supplementary video 1. COL4 supplies a scaffold for COL1 deposition in liver sinusoids to promote sinusoidal resistance. To illustrate the co-localization of COL4 and COL1 in liver sinusoids, liver sections from *Col4a1*$^{fl/wt}$ and *Col4a1*$^{fl/wt}$/*Cdh5*^{CreERT2} mice underwent CCl_4 administration were visualized by LSM980 Airyscan microscope and reconstructed the videos via Imaris analysis software after IF staining. Increased COL4 and COL1 in liver sinusoids after CCl_4

treatment were distributed differently. COL1 was structurally surrounded and supported by COL4. However, sinusoidal COL1 showed a patchy and discontinuous pattern without structural support of COL4 in *Col4a1*$^{fl/wt}$/*Cdh5*^{CreERT2} liver sinusoids. Green indicates COL4 stained with anti-COL4 antibody; red, staining of COL1; blue, DAPI staining of DNA.

Supplementary Fig 1

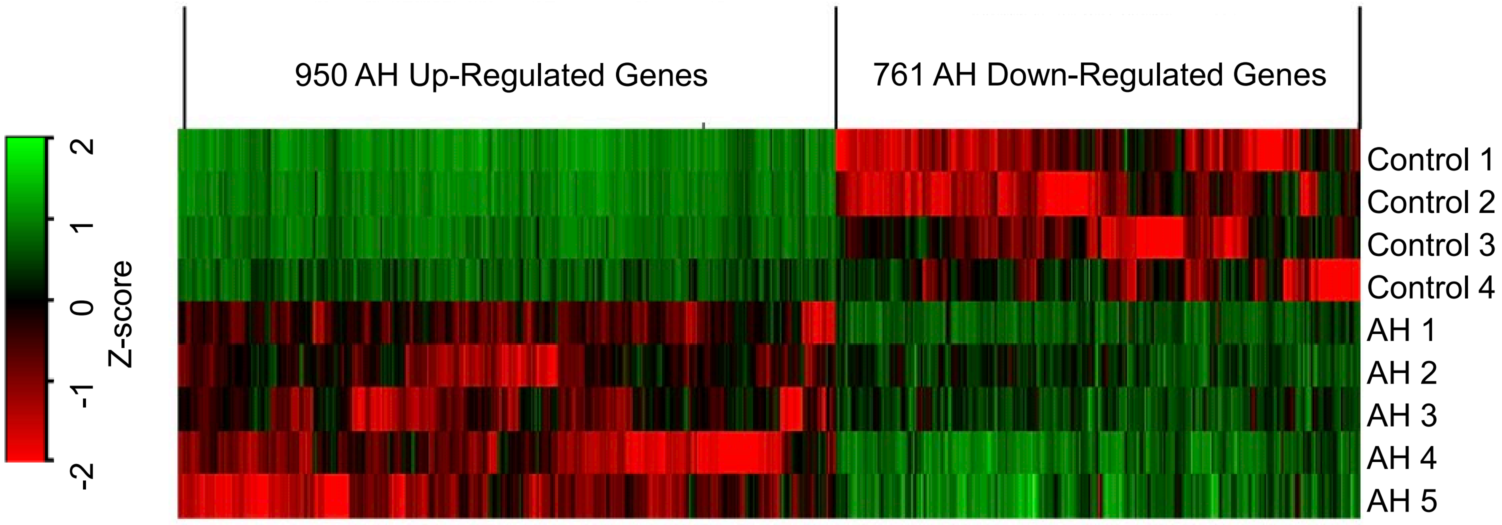

Supplementary Fig 2

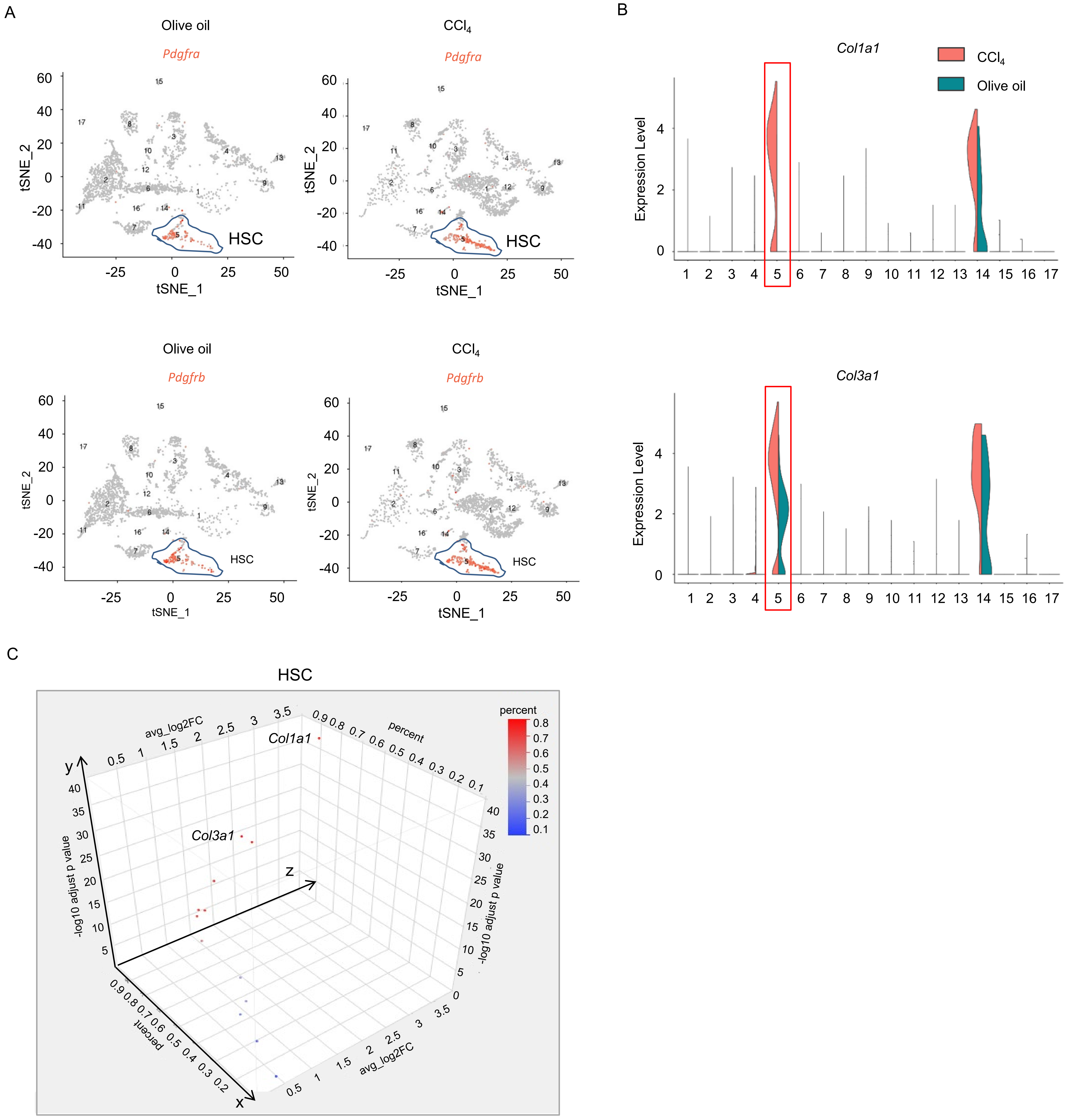

Supplementary Fig 2

D

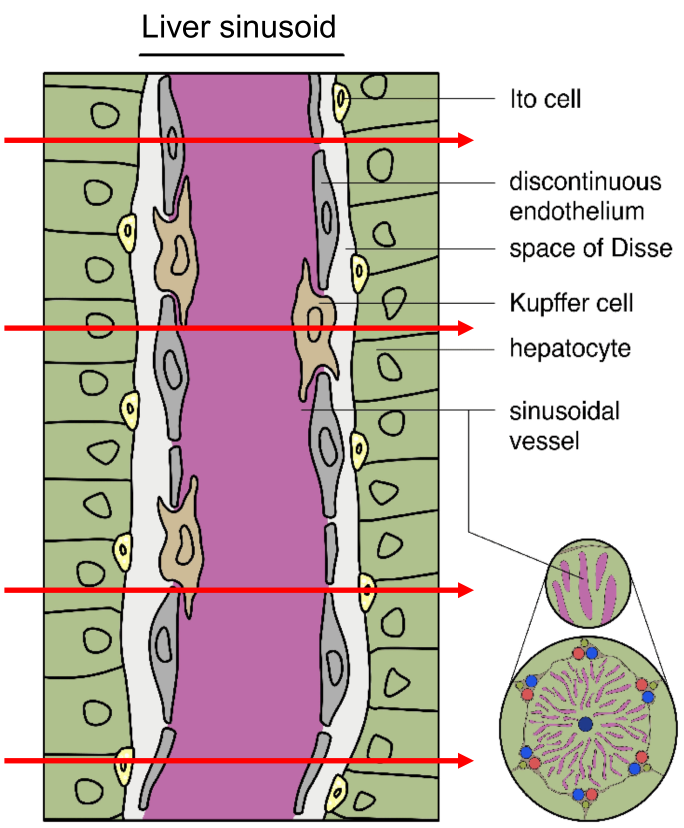

E

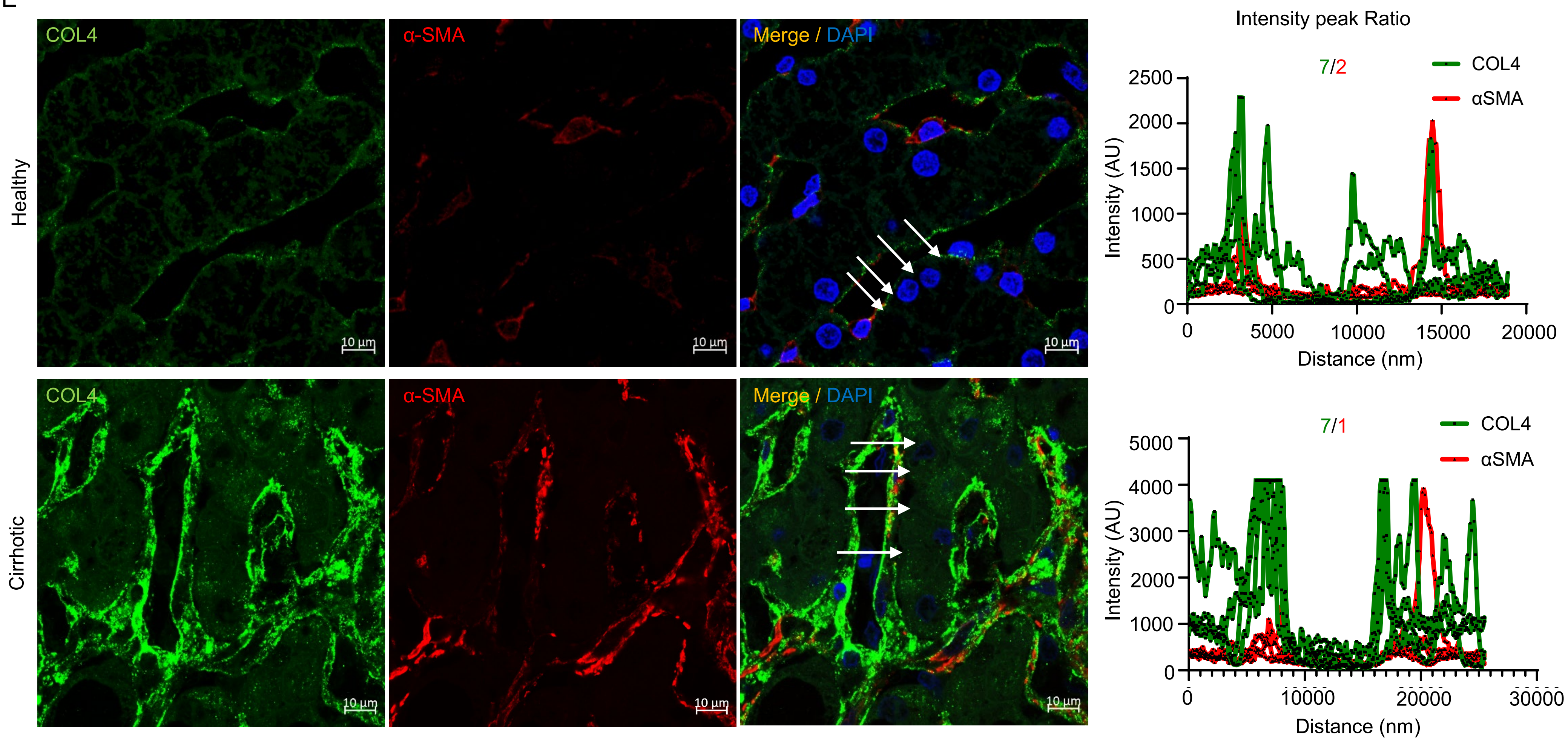

F

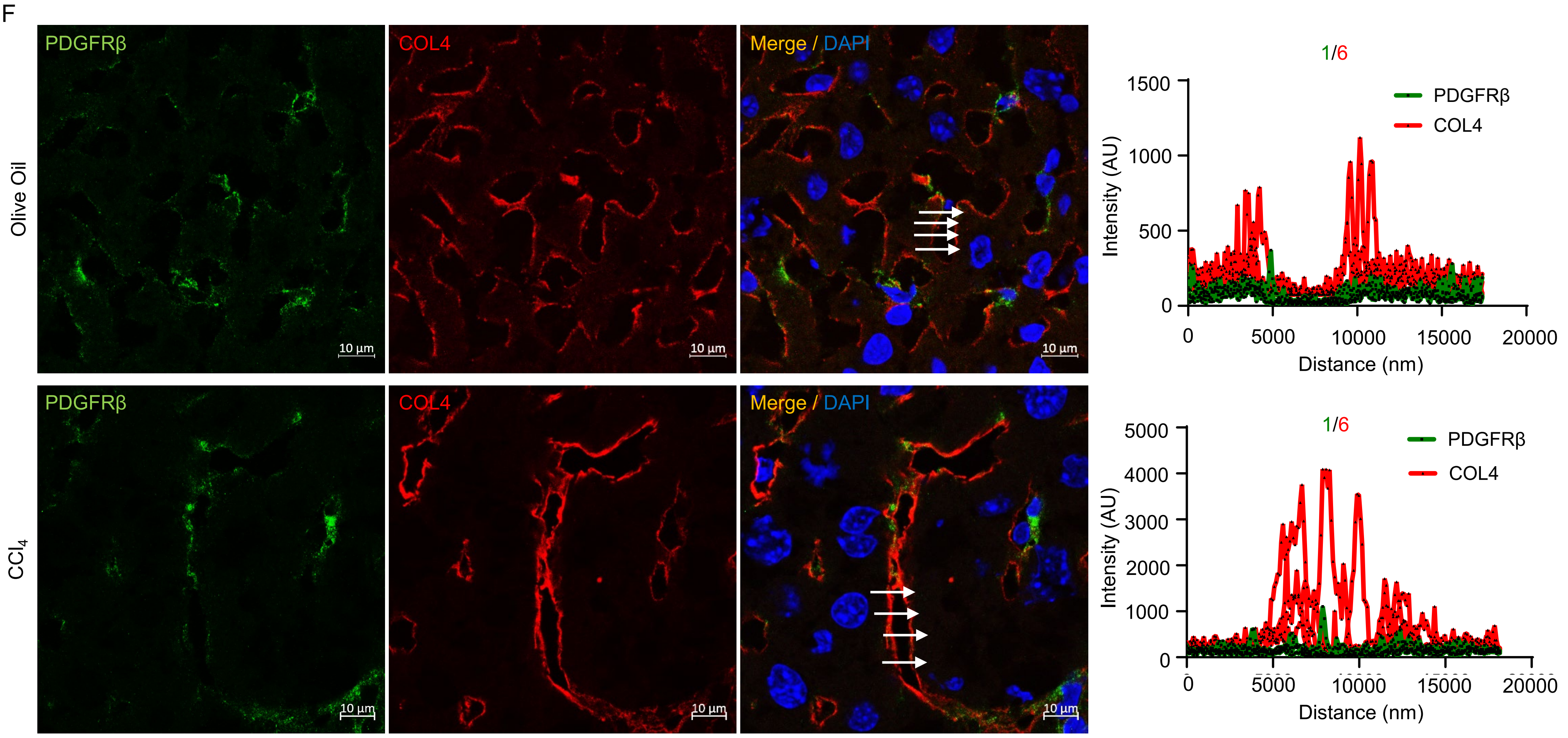

Supplementary Fig 3

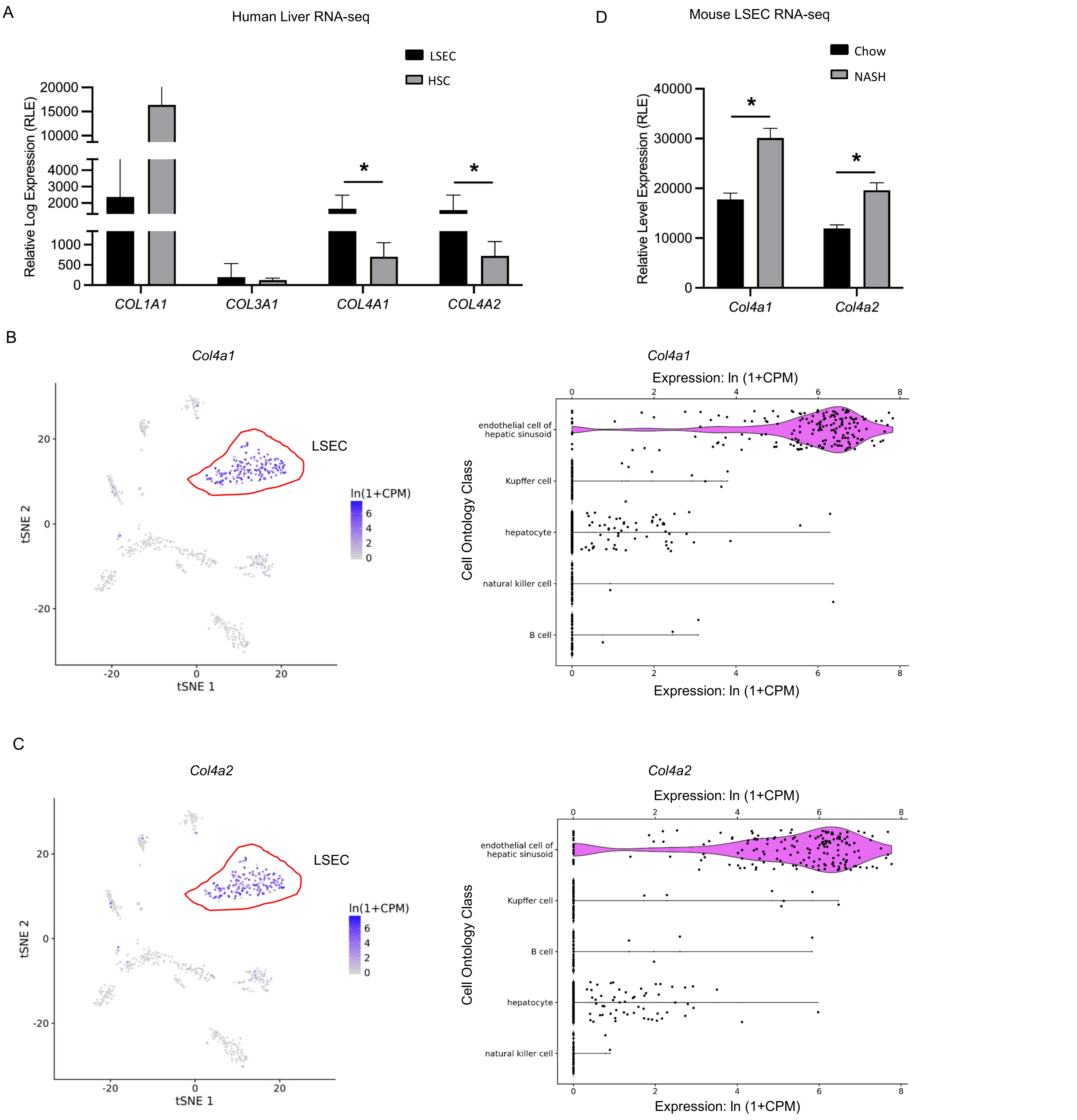

Supplementary Fig 4

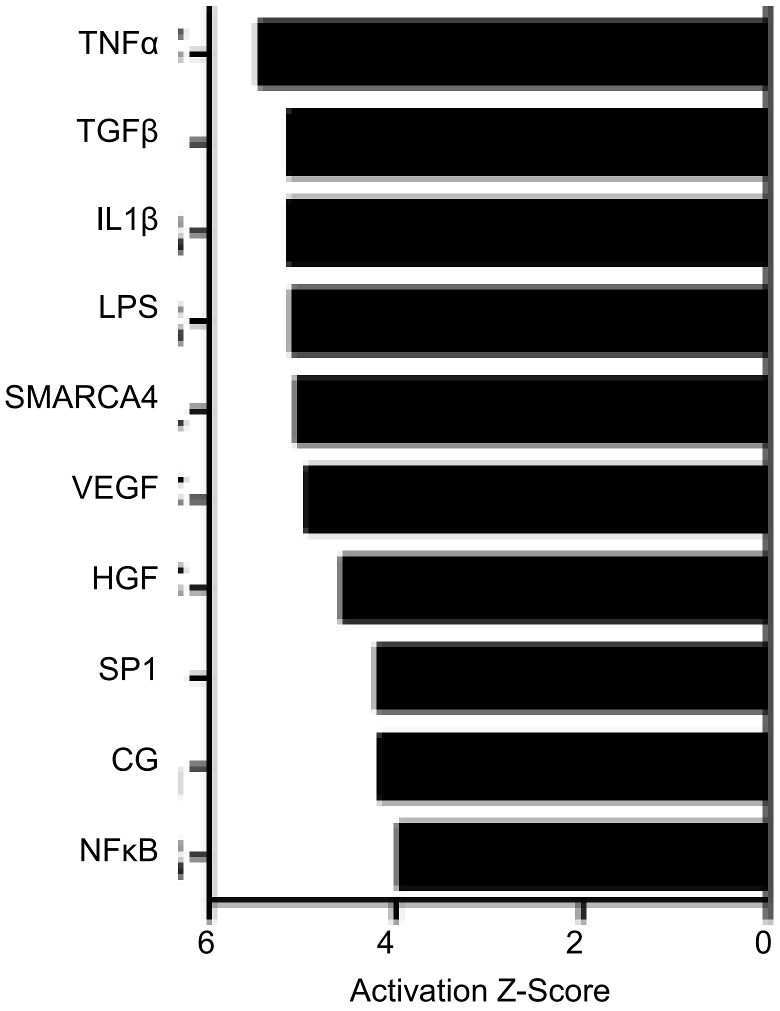

Supplementary Fig 5

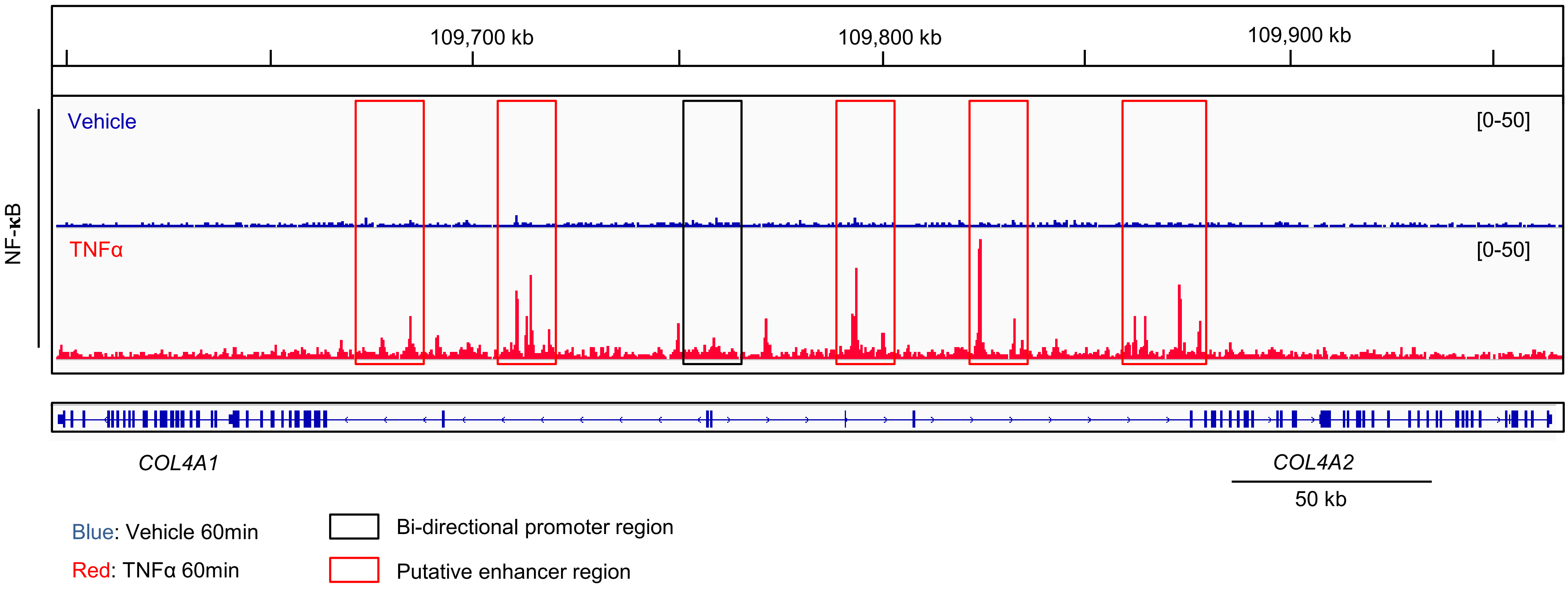

Supplementary Fig 6

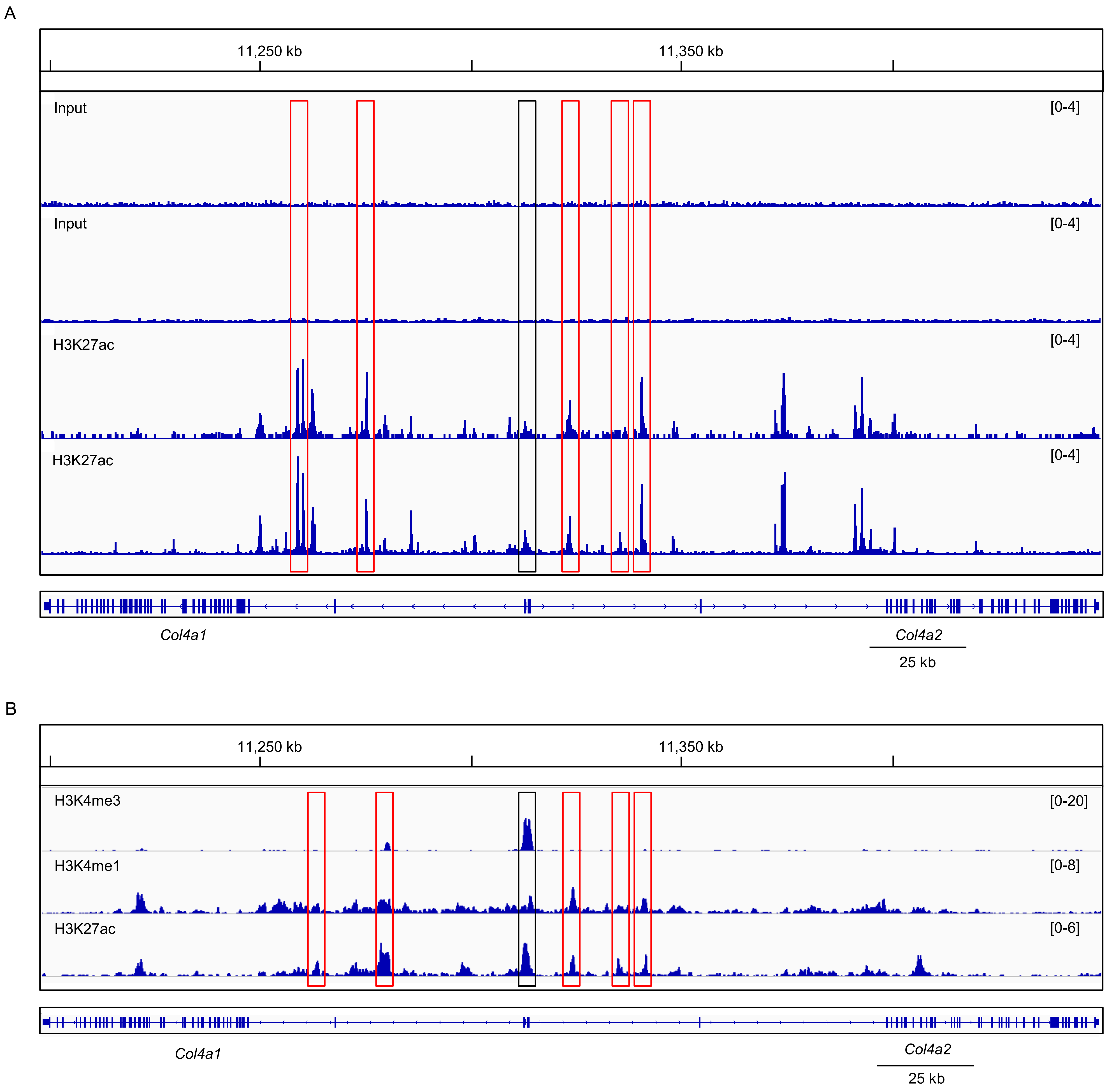

Bi-directional promoter region

Putative enhancer region

Supplementary Fig 7

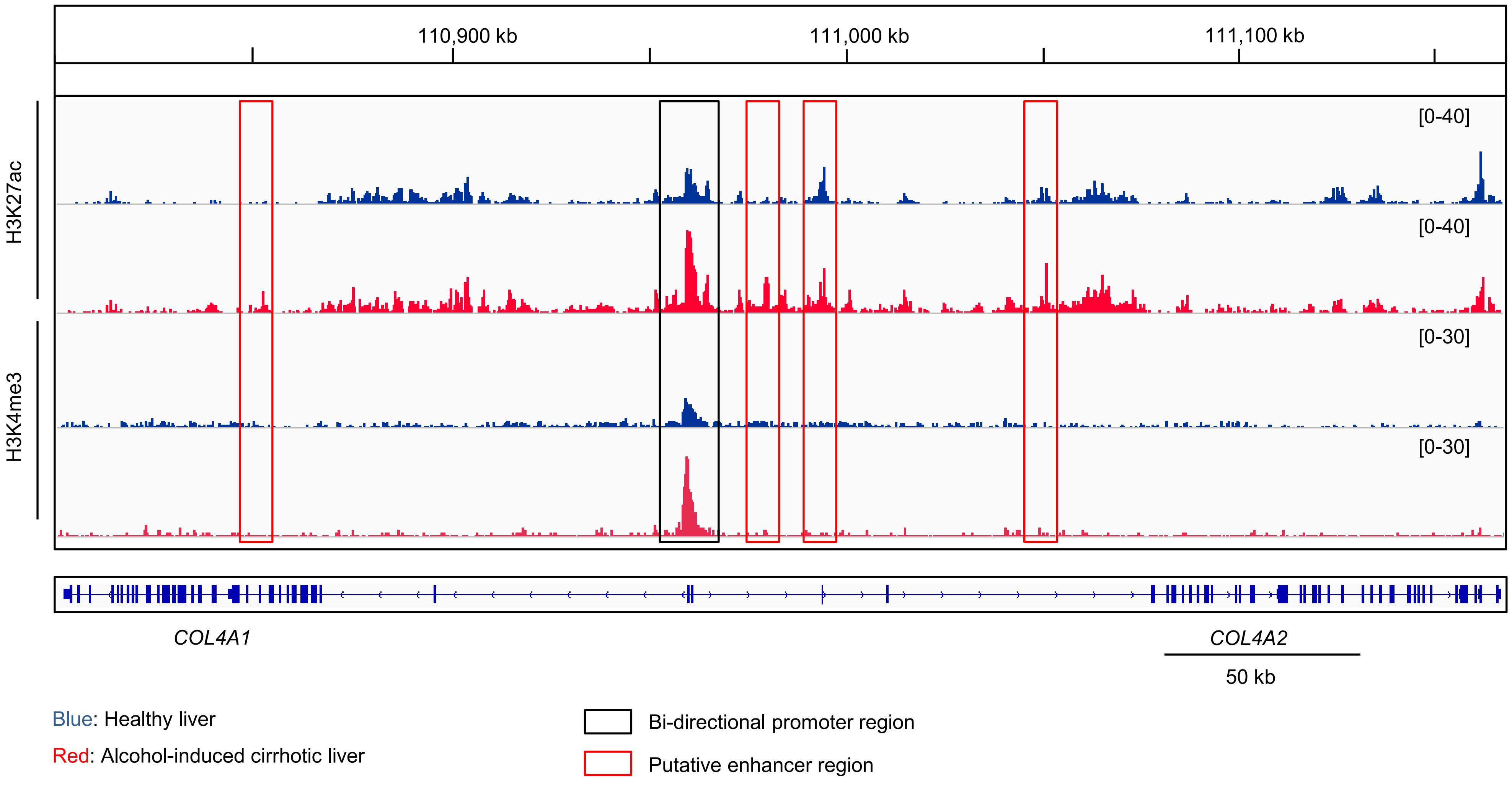

Supplementary Fig 8

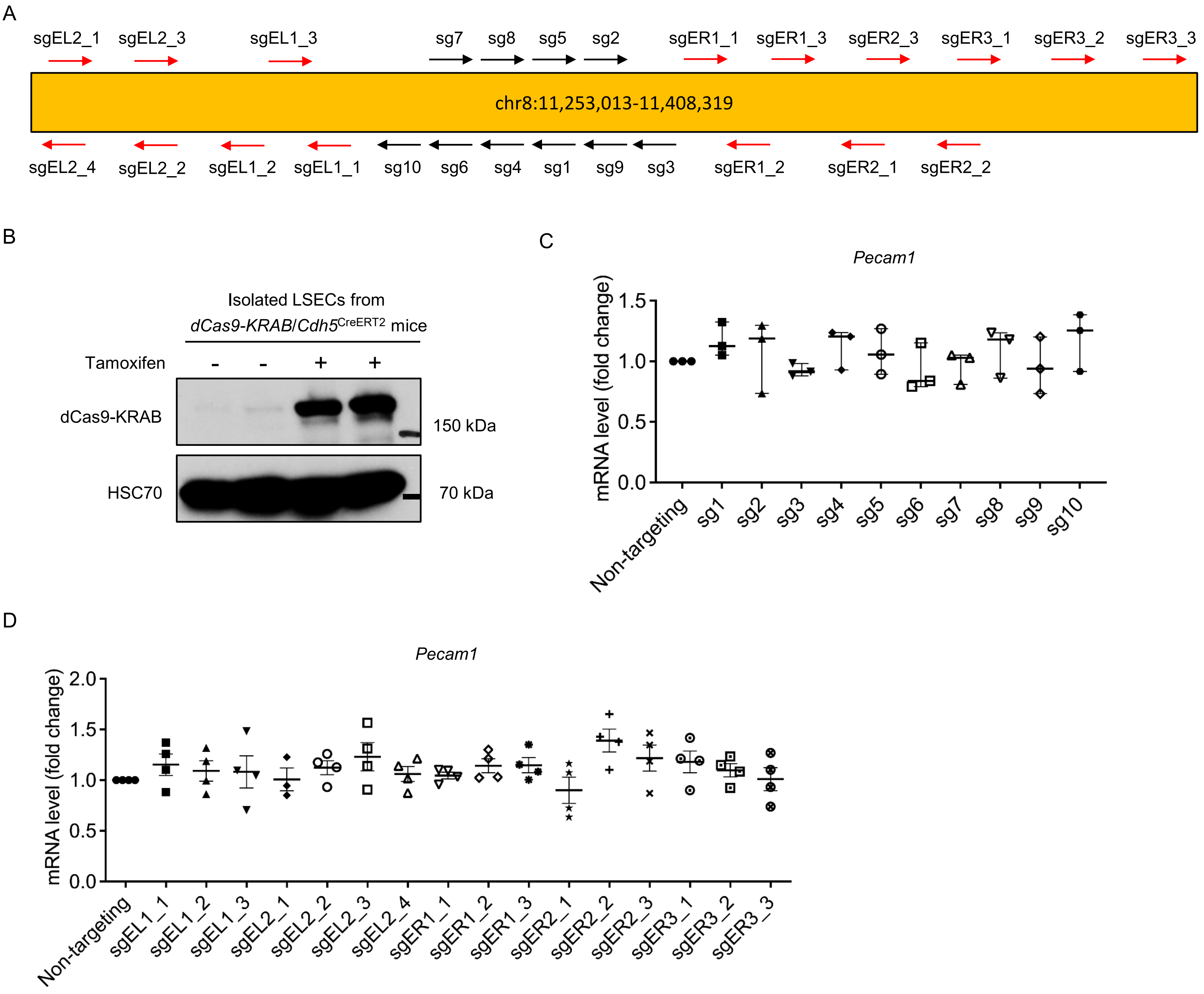

Supplementary Fig 9

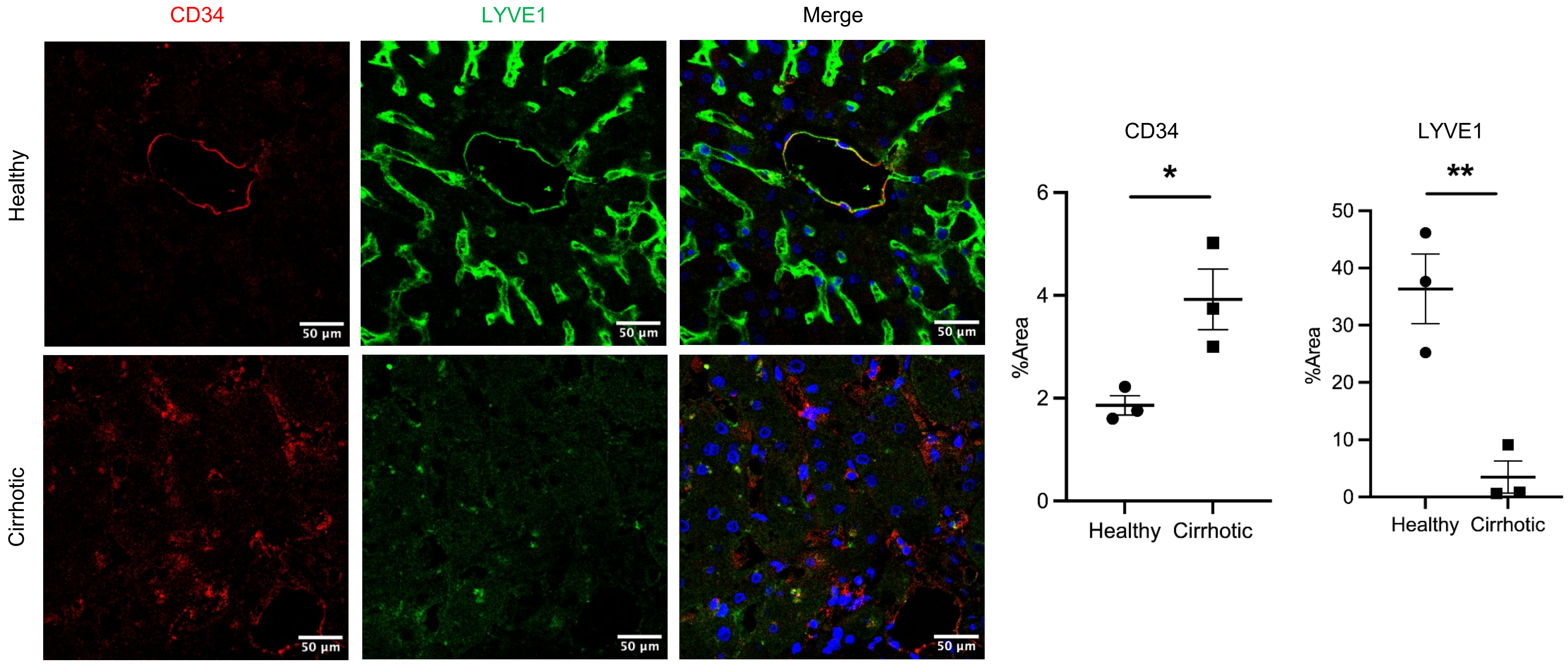

Supplementary Fig 10

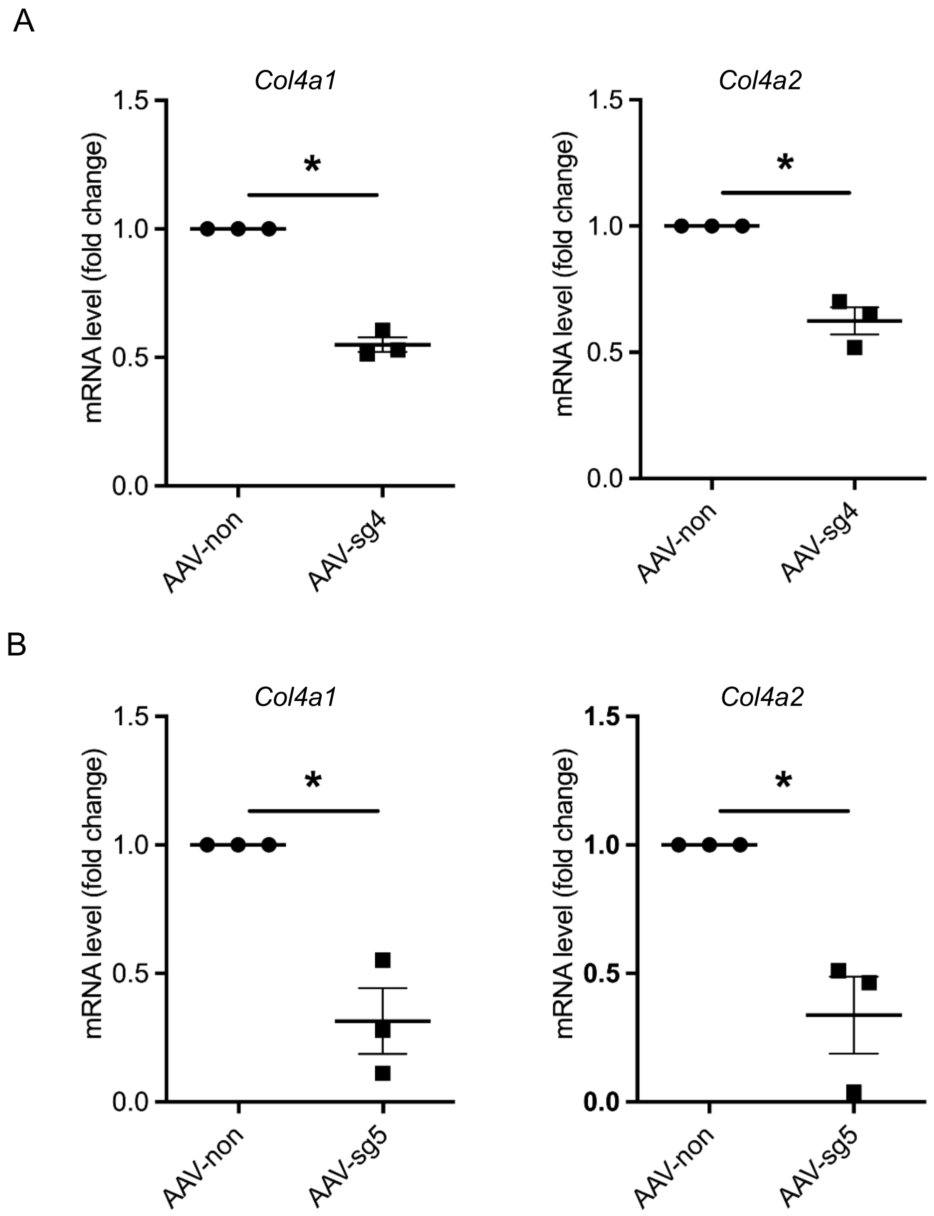

Supplementary Fig 11

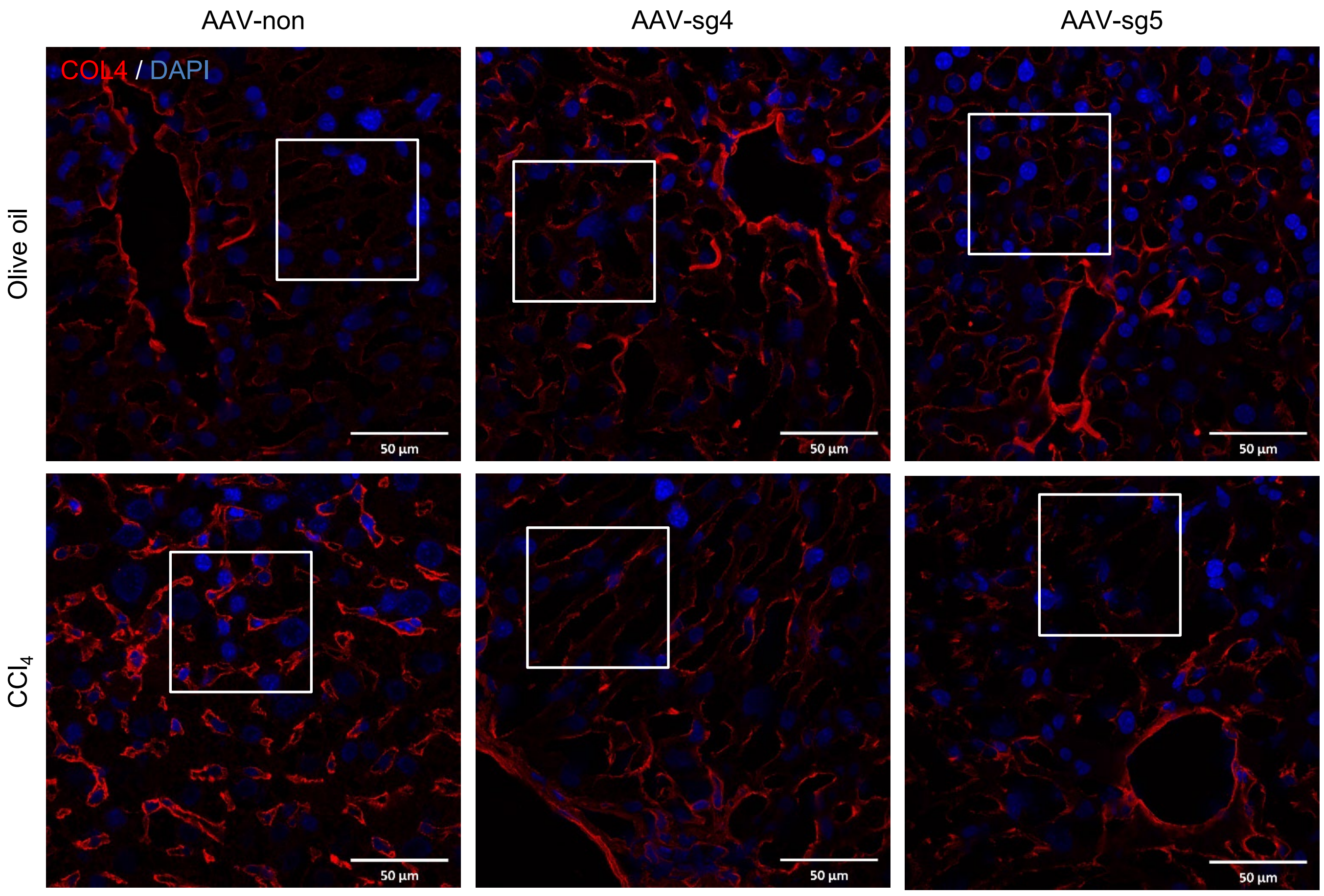

Supplementary Fig 12

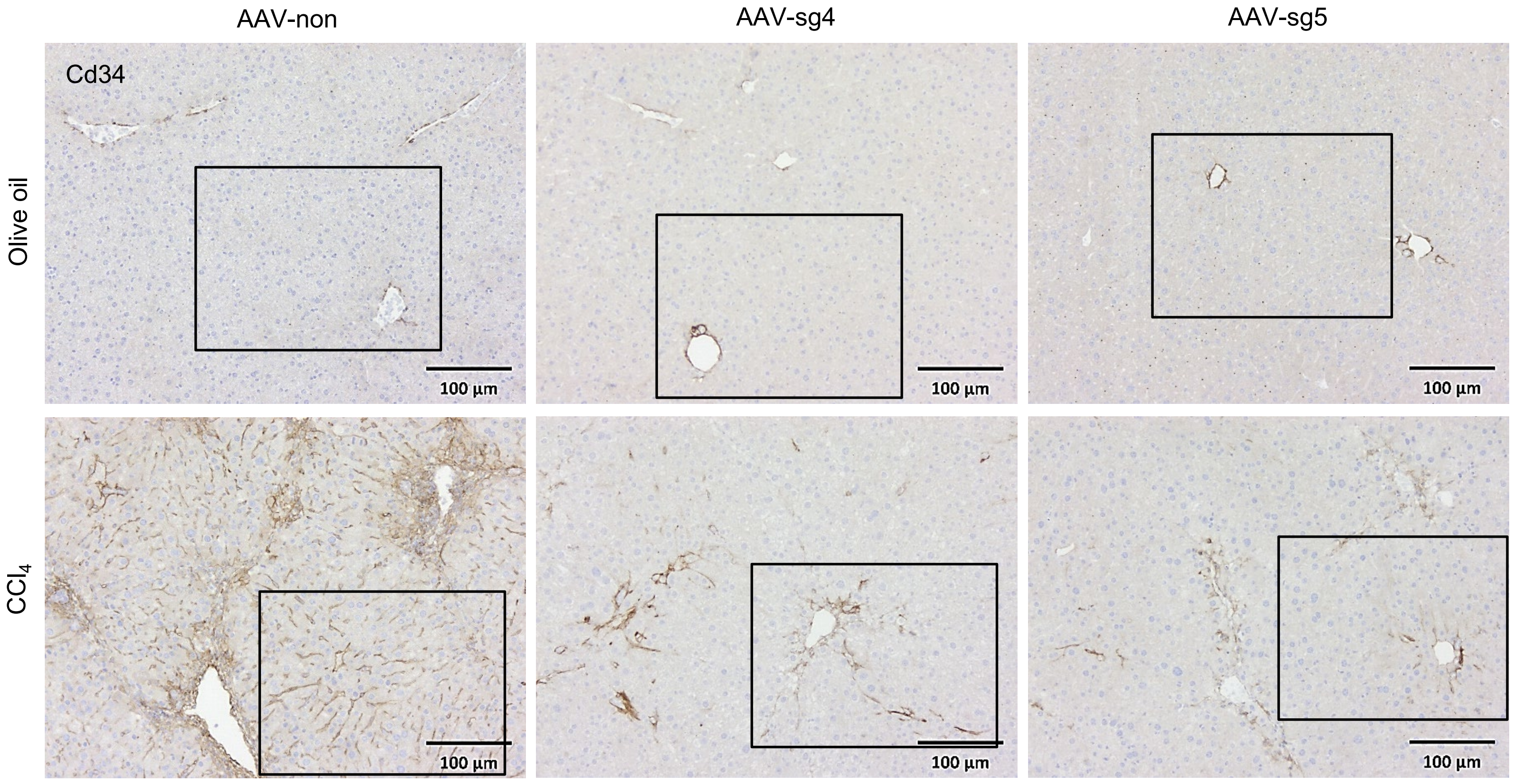

Supplement: Supplemental data [file jciinsight-9-174775-s198.pdf]
